# Antagonism of ALAS1 by the Measles Virus V protein contributes to degradation of the mitochondrial network and promotes interferon response

**DOI:** 10.1371/journal.ppat.1011170

**Published:** 2023-02-21

**Authors:** Pierre Khalfi, Rodolphe Suspène, Kyle A. Raymond, Vincent Caval, Grégory Caignard, Noémie Berry, Valérie Thiers, Chantal Combredet, Claude Rufie, Stéphane Rigaud, Amine Ghozlane, Stevenn Volant, Anastassia V. Komarova, Frédéric Tangy, Jean-Pierre Vartanian

**Affiliations:** 1 Virus and Cellular Stress Unit, Department of Virology, Institut Pasteur, Université de Paris Cité, Paris, France; 2 Sorbonne Université, Complexité du Vivant, ED515, Paris, France; 3 UMR1161 Virologie, ANSES-INRAE-ENVA, Maisons-Alfort, France; 4 Vaccines Innovation Laboratory, Institut Pasteur, Université de Paris Cité, Paris, France; 5 Image Analysis Hub, Institut Pasteur, Université de Paris Cité, Paris, France; 6 Bioinformatics and Biostatistics HUB, Institut Pasteur, Université de Paris Cité, Paris, France; 7 Interactomics, RNA and Immunity Laboratory, Institut Pasteur, Université de Paris Cité, Paris, France; NYU School of Medicine, UNITED STATES

## Abstract

Viruses have evolved countless mechanisms to subvert and impair the host innate immune response. Measles virus (MeV), an enveloped, non-segmented, negative-strand RNA virus, alters the interferon response through different mechanisms, yet no viral protein has been described as directly targeting mitochondria. Among the crucial mitochondrial enzymes, 5′-aminolevulinate synthase (ALAS) is an enzyme that catalyzes the first step in heme biosynthesis, generating 5′-aminolevulinate from glycine and succinyl-CoA.

In this work, we demonstrate that MeV impairs the mitochondrial network through the V protein, which antagonizes the mitochondrial enzyme ALAS1 and sequesters it to the cytosol.

This re-localization of ALAS1 leads to a decrease in mitochondrial volume and impairment of its metabolic potential, a phenomenon not observed in MeV deficient for the *V* gene. This perturbation of the mitochondrial dynamics demonstrated both in culture and in infected IFNAR^−/−^ hCD46 transgenic mice, causes the release of mitochondrial double-stranded DNA (mtDNA) in the cytosol. By performing subcellular fractionation post infection, we demonstrate that the most significant source of DNA in the cytosol is of mitochondrial origin. Released mtDNA is then recognized and transcribed by the DNA-dependent RNA polymerase III. The resulting double-stranded RNA intermediates will be captured by RIG-I, ultimately initiating type I interferon production. Deep sequencing analysis of cytosolic mtDNA editing divulged an APOBEC3A signature, primarily analyzed in the 5’TpCpG context. Finally, in a negative feedback loop, APOBEC3A an interferon inducible enzyme will orchestrate the catabolism of mitochondrial DNA, decrease cellular inflammation, and dampen the innate immune response.

## Author summary

As viruses evolve within the confines of an arms race with the host immune system, they develop means to evade or counteract detection by the immune system, thus facilitating their replication. In this light, many viruses have been described to encode proteins which target the mitochondrial network, a key hub in immune signaling. Previous work has highlighted the ability of MeV to interact with mitochondria, although the mechanism of perturbation remained elusive. Here we show that the MeV-V protein antagonizes the mitochondrial ALAS1 enzyme and relocalizes it to the cytosol, leading to an alteration of the mitochondrial network. We found that this disruption will induce a release of mitochondrial DNA (mtDNA), constituting a potential danger signal for the infected cell. By investigating the cascade of innate immune sensors triggered by mtDNA in the cytosol, we further show that the endogenous APOBEC3A cytidine deaminase is induced to target the released cytosolic mtDNA, attenuating the danger signal, and reducing cellular inflammation. Our study provides new mechanistic insight into the interaction of MeV with mitochondrial proteins, adding to the ever-growing repertoire of viral strategies to directly or indirectly target this organelle.

## Introduction

Viruses are subject to control by the innate immune system. Among the myriad of primary host defenses, programmed cell death and the interferon (IFN) system are particularly important in the battle against pathogens. During the relentless fight between viruses and the immune system, mitochondria are on the front line. Indeed, they function as a crucial signaling platform for various biological responses, including metabolism, innate immunity and inflammation.

Mitochondria are intracellular organelles considered to be the powerhouse of the cell, but they also play a central role in the primary host defense. Therefore, a key feature of some RNA or DNA viruses is to preferentially target the mitochondria, subsequently dampening the immune system’s response [1–5]. Mitochondria play a central role in key pathways of cellular metabolism, such as oxidative phosphorylation, calcium buffering and heme biosynthesis [6], each of which could be affected upon viral infections. The production and regulation of heme occurs through the precise control of the intracellular expression of 5-aminolevulinate synthase (ALAS1). This enzyme, encoded by a nuclear gene, catalyzes the condensation of glycine and succinyl-CoA to produce 5-aminolevulinic acid (ALA) in the mitochondrial matrix [7]. ALAS1 is the first, as well as the rate-limiting enzyme of the heme biosynthetic pathway in mammalian cells. Hence, it is likely that a heme deficiency leads to mitochondrial dysfunction. Indeed, analyses of mitochondria from skeletal muscle of aged wild-type and aged ALAS1 heterozygous mice indicated that ALAS1 deficiency was associated with a perturbed mitochondrial morphology [8].

Measles virus (MeV), an enveloped, non-segmented, negative-strand RNA virus, belonging to the family *Paramyxoviridae*, is known to differ in its IFN antagonistic ability. The MeV *P* gene is translated into three proteins: the phosphoprotein P (MeV-P), an essential polymerase cofactor, and two virulence factors the V (MeV-V) [9] and the C (MeV-C) proteins, which have been reported to possess multiple functions [10,11]. MeV-P and MeV-V proteins have been reported to antagonize type I and/or type II IFN pathways, although the precise mechanism of inhibition at the molecular level is not fully defined, largely due to a lack of direct analysis of the underlying molecular interactions [12–14].

Among the myriad of cytosolic DNA sensors, it has recently been shown that cytosolic mtDNA can be sensed and transcribed by RNA polymerase III [15–18]. This event, producing double-stranded (ds) RNA intermediates can be captured by RIG-I and lead to type I IFN production [18]. Type I IFN exhibits antiviral activity through its ability to induce IFN-stimulated genes (ISGs), such as the APOBEC3 (A3) cytidine deaminases, which are known to play major roles in viral restriction. The *A3* locus comprises a series of seven genes encoding six functional endogenous cytidine deaminases (A3A-A3C and A3F-A3H) [19–23]. All A3 enzymes, except A3DE, catalyze the deamination of cytidine to uridine on single-stranded DNA (ssDNA) which can be of viral, mitochondrial or nuclear origin, leading to CG->TA editing. A3 enzymes preferentially edit a cytidine residue in the 5′TpC, with the exception of A3G, which prefers 5′CpC dinucleotides [23–26].

Our studies demonstrate that during MeV infection, MeV-V protein antagonizes the mitochondrial enzyme ALAS1 and sequester it to the cytosol. This event will indirectly modulate the mitochondrial metabolism resulting in the fragmentation and degradation of the mitochondrial network, leading to the release of mtDNA in the cytosol. Mitochondrial dsDNA will then be detected and transcribed by DNA-dependent RNA polymerase III, resulting in dsRNA intermediates that will be captured by RIG-I, ultimately generating type I IFN production. In turn, A3A cytidine deaminase induction will lead to mtDNA editing, dsDNA breaks and apoptosis.

## Results

### MeV infection upregulates type I interferon and leads to *APOBEC3A* expression

**Fig 1:**
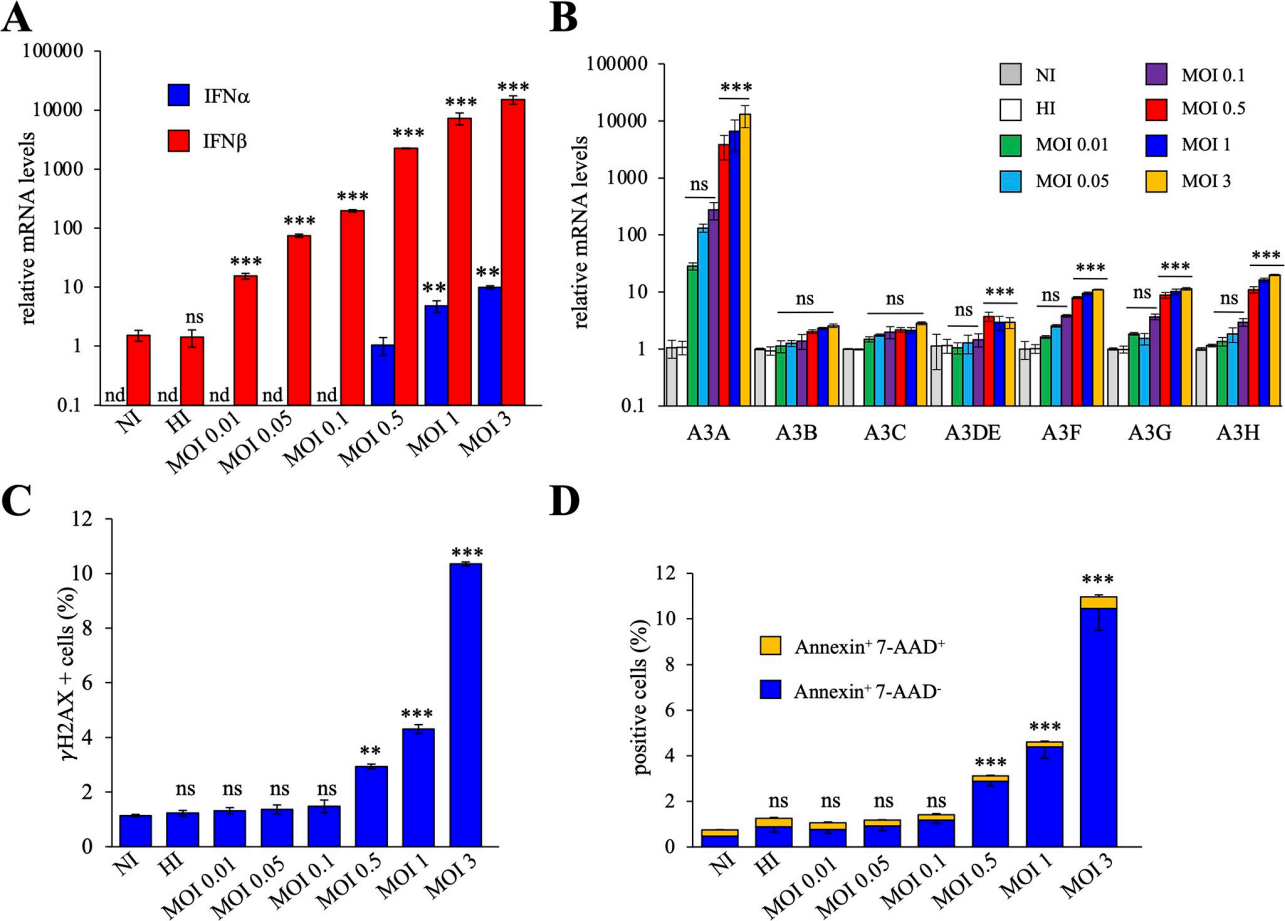
MeV induced *APOBEC3A* upregulation. A) Relative expression of *IFN-α* and *IFN-β* following MeV infections at different MOIs at 24 hpi. Gene expression was normalized using the expression level of the housekeeping gene *RPL13A*. NI: non-infected cells, HI: cells infected with heat inactivated virus. B) *A3* relative expression upon MeV infection in THP-1 cells for 24 hpi at different MOIs. A-B), mean values and standard deviations of the mean (s.e.m.) were calculated for three independent infections in duplicate (n = 6), unpaired two-side Student’s t-test, **, p < 0.05 and ***, p < 0.005, ns: not statistically significant, nd: not determined. C) Flow cytometry analysis of the γ-H2AX positive cells at 18 hpi by MeV at different MOIs. D) Flow cytometry analysis of the Annexin V positive cells at 18 hpi by MeV at different MOIs. C-D), mean values and s.e.m. were calculated for three independent infections (n = 3), unpaired two-side Student’s t-test, **, p < 0.05 and ***, p < 0.005, ns: not statistically significant.

To investigate the effects of viral stress on the expression of ISGs, we used the Schwarz live-attenuated vaccine strain of MeV, (GenBank accession no. AF266291.1) [27]. To optimize the conditions of infection, THP-1 cells were infected with MeV at a multiplicities of infection (MOI) 0.01, 0.5, 0.1, 1 and 3. The IFN-inducible THP-1 human acute monocytic leukemia cell line was chosen as it is permissive to MeV infection and facilitates viral replication [18]. At 24 hours post-infection (hpi), total RNA was extracted and *IFN-α* and *IFN-β* expressions were quantitated by RT-qPCR. As observed in Fig 1A, different MOIs of MeV generated strong *IFN-β* responses in a dose dependent manner, a ~15-15000-fold increase when compared to non-infected (NI) cells and cells infected with heat inactivated virus (HI) but failed to induce potent induction of *IFN-α* expression.

A transcriptional study of all *A3* genes showed that an increase in MeV MOI was correlated with *A3A* expression in a dose dependent manner by ~2–4 logs (Fig 1B), as well as a slight increase of *A3B*, *A3C*, *A3DE*, *A3F* and *A3G* expression by ~ 2-20-fold. When compared to other *A3* genes, *A3A* expression was by far the most sensitive to MeV infection (Fig 1B). Interestingly, as observed by γ-H2AX assays using flow cytometry and Western Blot (WB) (Fig 1C, S1A Fig), only MOI 0.5, 1 and 3 of MeV generated double-strand DNA breaks (DSBs) in THP-1 cells, ~2.5-9-fold above background (NI cells). DSBs were also correlated with induction of apoptosis as assessed using Annexin V and 7-AAD staining (Fig 1D). Together, these findings highlight a mechanism by which IFN-β preferentially induces the A3Α enzyme, which in turn induces DSBs and apoptosis in MeV infected cells.

### MeV induces the release of mitochondrial DNA into the cytosol *in vitro* and *in vivo*

To compare *IFN* and *A3A* expression upon infection, MeV and a recombinant V-defective MeV (MeV-ΔV) [28] were used at MOI 1 and 3 to infect THP-1 cells. To have an identical input, MeV and MeV-ΔV viruses were quantified by RT-qPCR and both viral titers were established by plaque assays (not shown). To compare the replication kinetics of MeV and MeV-ΔV, Vero and THP-1 cells were infected at MOI 0.1 and harvested 12, 24, 36, 48, 60 and 72 hpi (S2A and S2B Fig). Viral loads were quantified at each time-point by RT-qPCR, while virus titers were characterized by limiting dilution. We observed that the growth kinetics for MeV and MeV-ΔV viruses were similar at 24 hpi, with a slight decrease in the growth of MeV-ΔV viruses in THP-1 cells after 36 hpi (S2A and S2B Fig).

**Fig 2:**
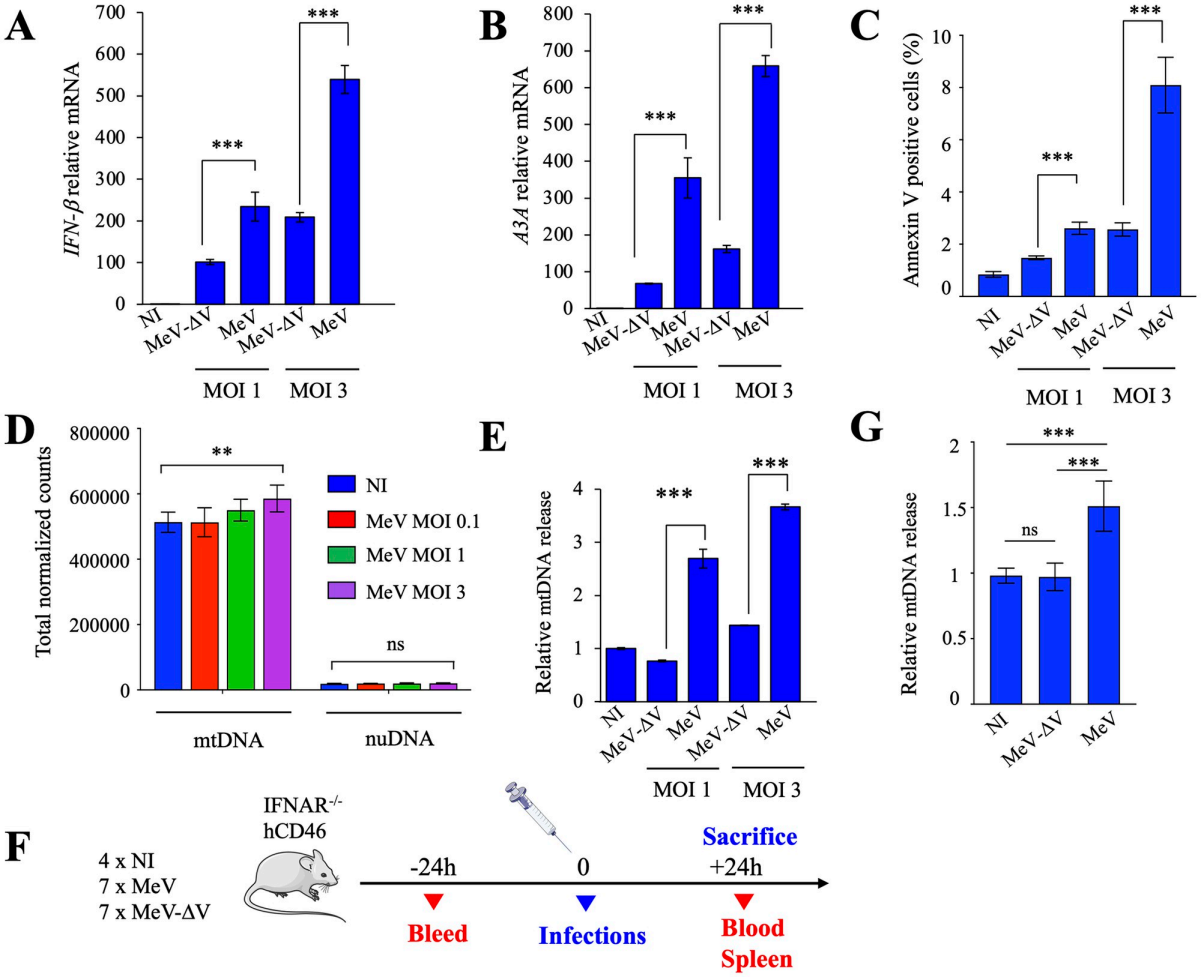
Cytosolic mitochondrial DNA release upon MeV and MeV-ΔV infection in culture and in mice. A-B) Relative expression of *IFN-β* and *APOBEC3A* following MeV-ΔV or MeV infections at MOI 1 and 3 at 24 hpi. Gene expression was normalized using the expression level of the housekeeping gene *RPL13A*. C) Flow cytometry analysis of the Annexin V positive cells at 24 hpi by MeV-ΔV or MeV at MOI 1 and 3. A-C), Mean values and s.e.m. were calculated for three independent infections (n = 3) (unpaired two-side Student’s t-test, ***, p < 0.005). D) Whole genome amplification of cytosolic DNA upon infection of MeV at MOI 0.1, 1 and 3 and blast to the human genome. E) THP-1 cells were fractioned at 24 hpi and mtDNA quantification was performed using the *MT-COI* gene in the cytosolic fraction and normalized to the quantification of the nuclear gene *β2M* of the total fraction. Mean values and s.e.m. were calculated for three independent infections in duplicate (n = 6) (unpaired two-side Student’s t-test, ***, p < 0.005 and ns: not statistically significant). F) IFNAR^−/−^ hCD46 mice were infected by intraperitoneal injection with 1 × 10^5^ TCID_50_ of MeV (n = 7), MeV-ΔV (n = 7) or 100μl PBS, (n = 4). At -24h and 24h after infection, blood and spleen were collected. G) CD11b^+^cells from infected mice were fractioned and mtDNA quantification was performed using the *MT-COI* gene in the cytosolic fraction and normalized to the quantification of the nuclear gene *β2M* of the total fraction. Unpaired two-side Student’s t-test, ***, p < 0.005 and ns: not statistically significant.

At 24 hpi, THP-1 infected cells were lysed, total RNA was extracted and *IFN-β* and *A3A* expressions were quantitated by RT-qPCR. As observed in Fig 2A and 2B, when compared to MeV-ΔV at MOI 1 and 3, MeV generated a ~2.5-fold increase in *IFN-β* and a ~4-5-fold increase in *A3A* response. Apoptosis induction was also ~1.7-3-fold lower during MeV-ΔV infection when compared to MeV as assessed by Annexin V staining (Fig 2C). These results suggest that possible alterations on mitochondrial membrane permeability, which have profound impact on mitochondrial bioenergetics, might be related to MeV-induced cell death and modulated by MeV-V protein.

As mitochondria is a sensitive organelle during viral infections, we next examined the involvement of cytosolic mtDNA sensors in the IFN signaling pathways. To demonstrate the relevance of cytosolic DNA sensors in stress signaling pathways, and to highlight the origin of cytosolic DNA, THP-1 cells were infected with MeV at MOI 0.1, 1 and 3. At 12 hpi, total DNA from cytosolic extracts were amplified by whole genome amplification, using random hexamers. As observed in Fig 2D, mtDNA was the most significant source of DNA detected in the cytosol, which was ~30-fold more abundant than nuclear DNA. Furthermore, when compared to NI, MeV infection at MOI 3 induces ~1.2-fold more mtDNA in the cytosol (Fig 2D).

To demonstrate the implication of MeV-V protein in the release of mtDNA in the cytosol, quantification was performed by RT-qPCR on *MT-COI* (mitochondrial cytochrome c oxidase subunit I). As observed in Fig 2E, MeV generated a ~2.5–3.5-fold increase of mtDNA in the cytosol at both MOI 1 and 3 when compared to MeV-ΔV. As a control, we verified the impact of the pro-apoptotic proteins Bax/Bak on the release of mitochondrial DNA. As observed in S1B Fig, Bax/Bak are not implicated on the impairment of the mitochondrial network.

To validate the effect of MeV and MeV-ΔV *in vivo*, we used IFNAR^−/−^ hCD46 transgenic mice, which are susceptible to infection by MeV and MeV-ΔV [29]. Animals were intraperitoneally injected with 10^5^ TCID50 of either MeV or MeV-ΔV or PBS as control at day 0 (Fig 2F). Blood was collected 24 hours before infection, and mice were sacrificed 24 hours after infection, spleen and blood were collected and CD11b^+^ cells were isolated. As visualized in Fig 2G, when compared to MeV-ΔV and NI, MeV generated a ~1.5-fold increase in cytosolic mtDNA. MeV and MeV-ΔV replication levels were similar as analyzed by SYBR RT-qPCR on the MeV *M* gene, prior and post-infection (not shown).

Altogether these data indicated that the source of cytosolic DNA is of mitochondrial origin, and MeV-V protein appears to be implicated in the mitochondrial network damages, allowing the release of mtDNA in the cytosol, evidenced in both cell culture and *in vivo*.

### MeV infection disturbs the mitochondrial network and cellular metabolism

**Fig 3:**
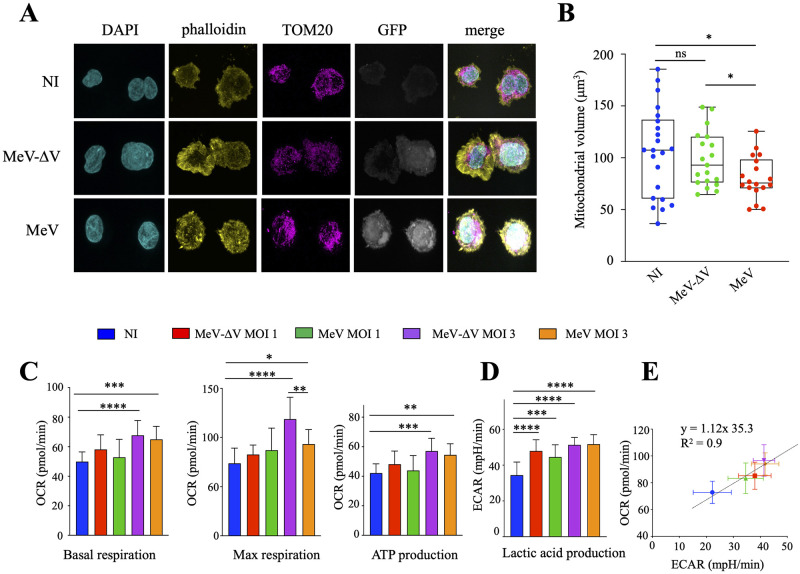
MeV disrupted mitochondrial network and cellular metabolism. A) Immunofluorescence of MeV-GFP infected cells at 24 hpi by confocal microscopy in Maximum Intensity Projection or segmented. Cells were labelled with a phalloidin high affinity filamentous actin probe (yellow), nuclei were stained using DAPI (blue), MeV-GFP or MeV-ΔV-GFP particles possess a GFP (grey) and mitochondria with an anti-TOM20 monoclonal antibody (purple). B) Total mitochondrial volume (MV) from confocal images. Box plots were statistically designed for 22 non-infected cells (NI), 18 MeV-GFP infected cells, and 19 MeV-ΔV-GFP infected cells. Infected MeV-GFP and MeV-ΔV-GFP cells were selected randomly based on the intensity of the GFP. Within the infected cell population, GFP negative cells were excluded from the analysis. Unpaired two-side Student’s t-test, *, p < 0.05, ns: not statistically significant). C) Measure of the oxygen consumption rate (OCR), the data obtained in S3B Fig were used to calculate the basal and max respiration and ATP production. D) The extracellular acidification rate (ECAR) was analyzed using the data obtained S3C Fig. E) Metabolic phenotype indicative of the bioenergetic state of mock- and MeV or MeV-ΔV-infected Vero cells (24 hpi) generated through OCR and extracellular acidification rate (ECAR) values under basal (normal) conditions and stressed conditions. C-D), data from the extracellular flux analysis were subjected to two-way ANOVA, followed by a Sidak post hoc test, *, p < 0.05, ** p < .01, ***, p < .001, ****, p < .0001, ns: not statistically significant.

To analyze different factors related to mitochondrial behavior following MeV infection, THP-1 cells were infected with MeV-GFP [27] and MeV-ΔV-GFP (both viruses encoding a green fluorescent protein) at MOI 3. At 24 hpi, cells were labelled with Alexa Fluor 555 phalloidin, a high affinity filamentous actin probe, mitochondria were stained with an anti-TOM20 antibody, and both viruses were visualized using the GFP encoded by the viral strain (Fig 3A). The analysis was done in maximum intensity projection (MIP), which corresponds to the maximum value along the depth axis (Fig 3A). For each MIP displayed, a corresponding segmentation is provided, which represents a visual of the segmentation result used to determine the volume of the mitochondria and the cells (S1C Fig).

When compared to the NI and MeV-ΔV-GFP infected cells, the mitochondrial network upon MeV-GFP infection appeared disorganized and less aggregated around the nucleus (Fig 3A and S1C Fig). To statistically validate these observations, images acquired by confocal microscopy were deconvoluted and parameters related to the total mitochondrial volume were analyzed. To calculate and compare the respective mitochondrial volume, 18 MeV-GFP^+^ and 19 MeV-ΔV-GFP^+^ infected cells were imaged and were compared to 22 randomly selected NI cells. As observed in Fig 3B, MeV-GFP infected cells exhibit 1.31 and 1.22-fold smaller mitochondrial network when compared to NI and MeV-ΔV-GFP, respectively (Fig 3B), and visualized in S1 (NI), S2 (MeV-ΔV-GFP) and S3 (MeV-GFP) movies. These results demonstrate that MeV-V protein is involved in altering the shape and volume of the mitochondrial network, leading to reduced and clustered mitochondria around the nucleus.

Replication kinetics of MeV-GFP and MeV-ΔV-GFP were analyzed in THP-1 and Vero cells, and were compared to MeV and MeV-ΔV viruses (S2C and S2D Fig). Viral loads were quantified at each time-point by RT-qPCR, while virus titers were characterized by limiting dilution. As observed with MeV and MeV-ΔV viruses, the growth kinetics for MeV-GFP and MeV-ΔV-GFP viruses were similar at 24 hpi, with a slight decrease for MeV-ΔV-GFP viruses on THP-1 cells (S2C and S2D Fig).

To assess the effect of the virus on cellular bioenergetics, Vero cells were infected with MeV and MeV-ΔV at MOI 1 and 3. At 16 hpi, an analysis of mitochondrial extracellular fluxes was performed using the Seahorse extracellular flux analyzer (S3A and S3B Fig). We evaluated the oxygen consumption rate (OCR) and the extracellular acidification rate (ECAR), as both are representative indicators of mitochondrial oxidative phosphorylation system (OXPHOS) and lactic acid production, respectively.

As observed in Fig 3C and S3C Fig, MeV as well as MeV-ΔV induced an increase in basal and maximal mitochondrial respiration and ATP production, with a more pronounced effect for MeV-ΔV. Indeed, a 1.3 and 1.8-fold increase of maximal respiration and spare respiratory capacity with MeV-ΔV, compared to MeV at MOI 3 was observed. Lactic acid measurement (ECAR, Fig 3D and S3D Fig) was also increased with both MeV and MeV-ΔV infections. The induced shift of the metabolic phenotype of MeV and MeV-ΔV-infected Vero cells to a higher energetic state was further visualized in a metabolic phenogram obtained through plotting the OCR against the ECAR values under basal and stressed conditions (Fig 3E). Infection with MeV and MeV-ΔV increased the OCR/ECAR ratio compared to NI in a dose dependent manner, which points toward an increase in mitochondrial metabolic activity (Fig 3E).

Altogether, these data indicate that MeV and MeV-ΔV both disrupt mitochondrial metabolism at high MOI, although a more significant increase of maximal respiration and spare respiratory capacity is observed during MeV-ΔV infection (Fig 3C, S3C Fig).

### Interaction of the mitochondrial enzyme ALAS1 with MeV-V protein

As MeV and MeV-ΔV damage the mitochondrial network differently, we decided to investigate further and identify the potential direct host interactors of MeV-V protein by performing a yeast two-hybrid screen (S4A Fig). Amongst a 2.10^7^ colonies screen derived from the human spleen cDNA library, the human *ALAS1* gene was recovered.

**Fig 4:**
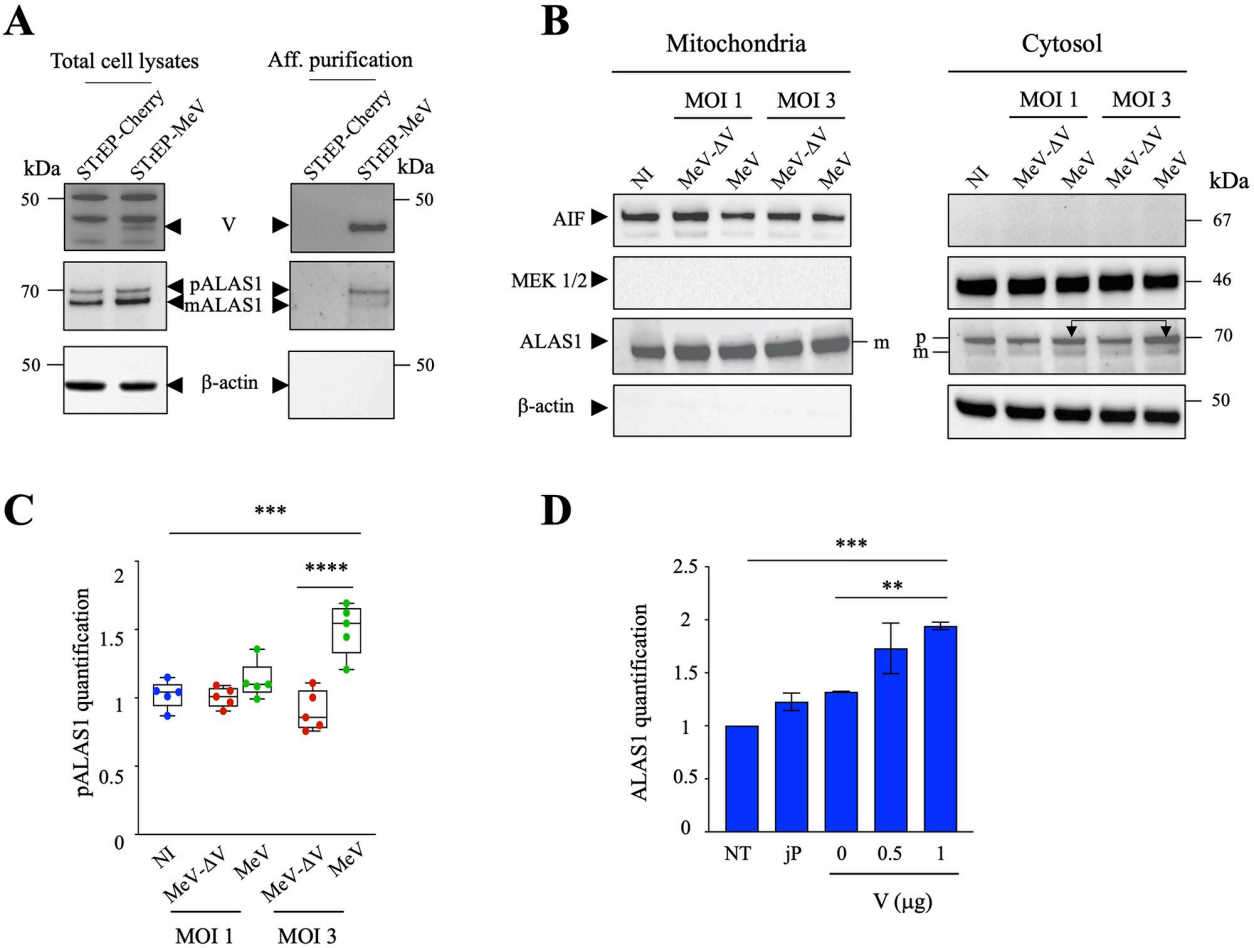
Mitochondrial enzyme ALAS1 interacts with MeV-V protein. A) THP-1 cells were infected with the MeV expressing the One-STrEP-tagged MeV-V protein or the One-STrEP-tagged CH protein used as control. Expression of One-STrEP-tagged MeV-V proteins were determined by Western blot in total cell lysates (left panel) or following affinity purification on Strep-Tactin Sepharose beads using a rabbit polyclonal anti-V rAb kindly provided by Dr. Kaoru Takeuchi [47] (right panel). ALAS1 protein was detected only with MeV expression of One-STrEP-tagged MeV-V proteins prior chromatography purification. B) Purity control of cellular fractions by immunoblotting, using β-actin to indicate the cytosol, AIF (apoptosis inducing factor) for mitochondria and MEK1/2 as a marker of cytosolic proteins. kDa: kilo Dalton; p: precursor, m: mature. Arrows represent the two forms of ALAS1. C) Quantification of ALAS1 protein upon MeV or MeV-ΔV infections at MOI 1 and 3. Mean values and s.e.m. were calculated for five independent experiments (unpaired two-side Student’s t-test, ***, p < 0.005). D) Quantification of ALAS1 protein upon transfection with 0, 0.5 and 1μg of MeV-V plasmid. NT: non transfected, jP: jetPRIME. Mean values and s.e.m. were calculated for two independent experiments. Unpaired two-side Student’s t-test, **, p < 0.05, ***, p < 0.005.

To validate the interaction of ALAS1 with MeV-V protein, we used a recombinant MeV with an additional copy of *V* ORF containing an amino-terminal One-STrEP Tag [30] (rMeV/STrEP-V). THP-1 cells were then infected with rMeV/STrEP-V or a negative control virus rMeV/STrEP-Cherry with MOI of 1 [30]. At 24 hpi, STrEP-V and STrEP-Cherry proteins were isolated by previously validated affinity purification using Strep-Tactin Sepharose beads [30]. To verify the accuracy of the affinity purification, Western blots performed on total cell lysates and lysates prepared after affinity purification were compared. As observed in Fig 4A, ALAS1 shows a strong interaction with One-STrEP-V. Furthermore, no interaction between ALAS1 and One-STrEP-Cherry was detected (Fig 4A, right panel).

It has been described that ALAS1 could be found in two forms, precursor (p) and mature (m) within the cells [31]. Following mitochondrial import, ALAS1 precursor (pALAS1) is proteolytically removed, giving rise to ALAS1 mature protein (mALAS1) that catalyzes the condensation of glycine and succinyl-CoA to produce 5-aminolevulinic acid in the mitochondrial matrix. Interestingly, pALAS1 isoform detected in the mitochondrial compartment was converted to mALAS1, with a relatively similar expression (Fig 4B left panel). Alternatively, in the cytosolic fraction, both p and mALAS1 were present, however with a higher amount of pALAS1, as was also visualized upon affinity purification using Strep-Tactin Sepharose beads (Fig 4A and 4B, right panels). When comparing infection of MeV at MOI 1 and 3, the amount of pALAS1 detected in the cytosol is increased in a dose dependent manner, by ~2-fold (Fig 4B, right panel with arrows and Fig 4C). Moreover, when analyzing MeV and MeV-ΔV infections, we observed that MeV leads to a higher amount of pALAS1 in the cytosol, demonstrating that MeV-V protein interacts with ALAS1 outside of the mitochondria (Fig 4B and 4C).

To ascertain that MeV-V protein sequestered ALAS1 in the cytosol, HeLa cells were transfected with increasing amounts of a plasmid encoding the V protein (0, 0.5 and 1μg). At 24 hours post transfection, whole cell extraction (WCE) was subjected to digitonin fractionation to obtain purified cytosolic extracts, and ALAS1 was quantitated. When comparing transfection of V with non-transfected (NT) cells or transfected cells only with jetPRIME, the amount of ALAS1 detected in the cytosol is increased in a dose dependent manner, by ~2-fold (Fig 4D).

Altogether, these results obtained by the two-hybrid screening and confirmed by STrEP-V purification from infected cells indicate that ALAS1 is an interactor of the MeV-V protein. This specific interaction orchestrates the localization of ALAS1 outside of the mitochondria, resulting in the damage of the mitochondrial network, and the subsequent release of mtDNA into the cytosol.

### Cytosolic mitochondrial DNA is the source of IFN production

To show that cytosolic DNA is the main inducer of type I *IFN-β* and *A3A* expressions, a stable THP-1 cell line expressing Deoxyribonuclease I (DNase I)-mCherry fusion protein and a catalytically inactive mutant DNase I_N7A_ [32] used as negative control were established. Expression of the DNase I-mCherry and DNase I_N7A_-mCherry were confirmed by Western blot (S4B Fig) and the cellular localization was assessed by confocal microscopy, demonstrating that DNase I and DNase I_N7A_ were exclusively detected in the cytosol (S4C Fig). Early apoptosis (annexin V^+^ 7-AAD^−^ cells) and late apoptosis/necrosis (annexin V^+^ 7-AAD^+^ cells) analysis showed that neither DNase I nor DNase I_N7A_ overexpression were toxic for the cells (S4D Fig).

To demonstrate that the DNase I-mCherry expressing THP-1 cells possess a functional enzymatic activity, cells were incubated for 24 hours with 20 μg/ml of peptidoglycans (PGN), known to stimulate IFN-α via TLR2 [33]. Quantification by RT-qPCR showed a ~60-90-fold induction of *A3A* expression (S5A Fig). The functionality of the DNase I-mCherry was validated by transfecting 250 ng of dsDNA into THP-1 cells expressing DNase I. When normalized to wild-type THP-1 cells, transfection of dsDNA generated a ~2-fold decrease in *A3A* responses (S5B Fig). These data demonstrate that the DNase I is enzymatically active and could induce DNA degradation, highlighting that the IFN and A3A pathways were unaltered.

To confirm that cytosolic DNA could enhance IFN and A3A productions, THP-1 expressing DNase I and DNase I_N7A_ were infected with MeV at MOI 1 or with a HI virus. At 24 hpi, *A3A* gene expression was quantitated by RT-qPCR. As seen in S5C Fig, *A3A* expression was reduced ~4-fold in THP-1 expressing DNase I, while in THP-1 expressing DNase I_N7A_ and THP-1cells, *A3A* expression remained constant. As a control, we can observe that neither NI nor HI conditions impacted *A3A* expression (S5C Fig). To demonstrate that the expression of DNase I in THP-1 did not disrupt MeV viral replication, SYBR RT-qPCR was performed on MeV *M* gene. As observed in S5D Fig, similar MeV *M* RNA expression was detected in THP-1 cell lines expressing DNase I or DNase I_N7A_ and in wild-type THP-1, indicating that MeV replication was not perturbed by the DNase I enzyme.

Taken together, these results demonstrate that cytosolic DNA plays a crucial role in priming the innate response. These findings illustrate that MeV infection leads to the release of mtDNA in the cytosol, triggering a robust innate antiviral immune response by inducing IFN-β, which in turn upregulates *A3A* expression.

### Cytosolic double-stranded DNA is transcribed by RNA polymerase III and impacts RIG-I

**Fig 5:**
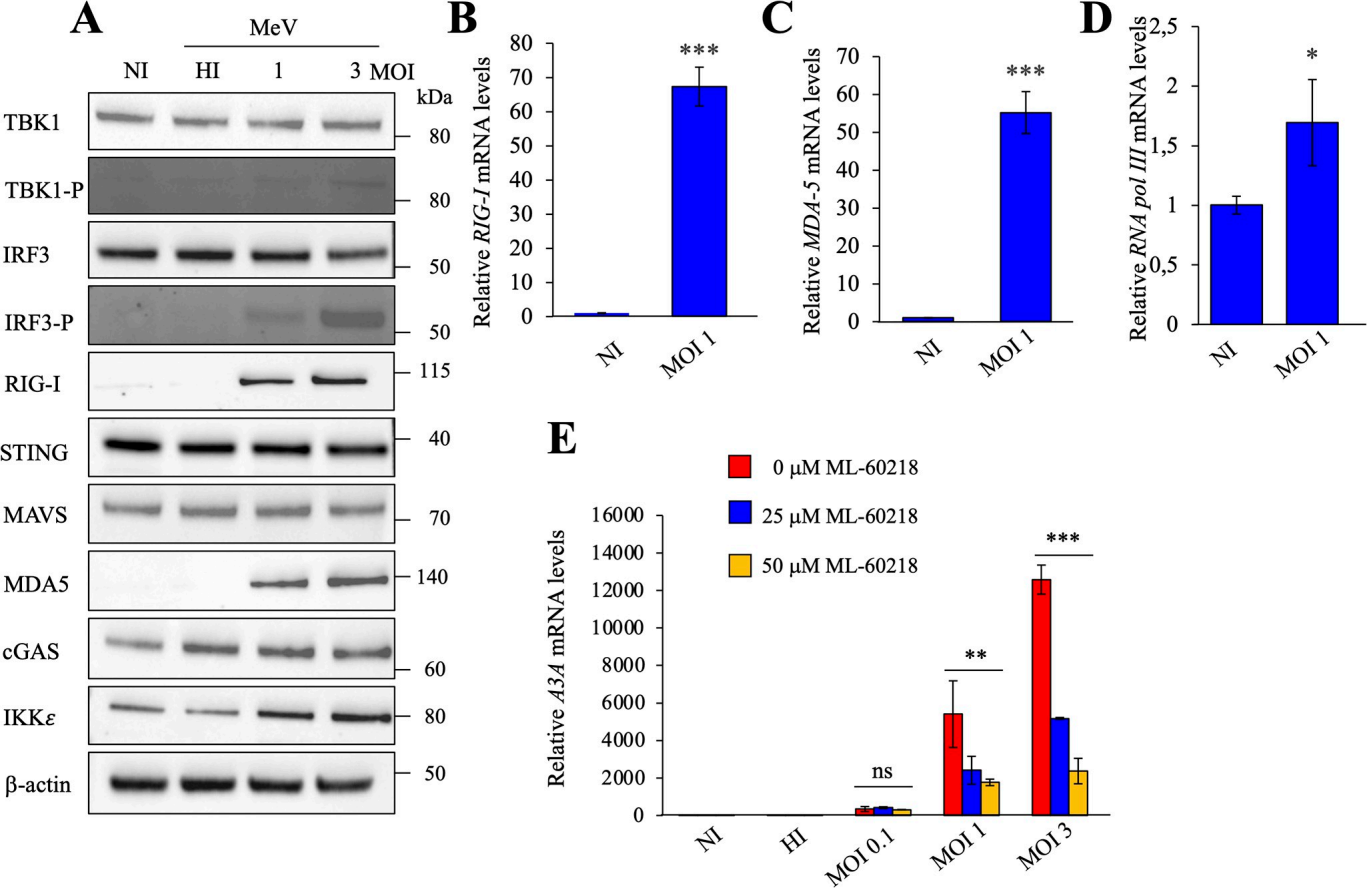
MeV induced RIG-I pathway *via* RNA polymerase III. A) Western blot analysis of TBK1, TBK1-P, IRF3, IRF3-P, RIG-I, MAD5, MAVS, STING, IKK*ε* and cGAS at 24 hpi by MeV at MOI 1 and 3 and compared to non-infected cells (NI) and cells infected by heat-inactivated virus (HI). β-Actin protein expression was used as loading control. kDa: kilo Dalton. B-D) Gene profiling of the IFN pathway after infection by MeV at 24 hpi: B) *RIG-I*, C) *MDA-5*, D) RNA polymerase III, expressions were normalized to *RPL13A* housekeeping gene expression level. Mean values and s.e.m. were calculated for three independent experiments in duplicate (n = 6). E) *A3A* relative expression, normalized to *RPL13A* housekeeping gene expression upon MeV infection at 0.1, 1 and 3 MOI and concomitant treatment with ML-60218 (inhibitor of RNA polymerase III) at 25 μM or 50 μM. Unpaired two-side Student’s t-test, **, p < 0.05, ***, p < 0.005 and ns: not statistically significant.

To identify the sensing molecules involved in the IFN pathway, THP-1 cells were infected with MeV at MOI of 1 and 3 and were compared to NI or HI virus. At 24 hpi, Western blot analysis showed increased levels of the phosphorylated forms of TBK1 (TBK1-P) and IRF3 (IRF3-P), as well as MOI-dependent increases in RIG-I, MDA5 and IKKε levels (Fig 5A). Steady state levels of TBK1, IRF3, STING and MAVS were unchanged. As observed in Fig 5A, dose dependent increases of TBK1-P and IRF3-P, key regulators of IFN production, suggest the involvement of STING and/or MAVS pathway. RT-qPCR performed on total RNA extracted from THP-1 cells infected by MeV at MOI 1 showed an upregulation of RIG-I and MDA5 expression of ~67 and 55-fold respectively over background levels (Fig 5B and 5C). Interestingly, we also noticed a slight ~1.7-fold increase of RNA polymerase III expression when compared to NI cells (Fig 5D), suggesting a role of this enzyme in the process. As RNA polymerase III has been implicated in the transcription of cytosolic dsDNA [15,17], we further explored this pathway.

To demonstrate that RNA polymerase III is an essential component of the multi-step IFN induction process, THP-1 cells were infected with MeV at MOI 0.1, 1 and 3 and incubated during 24 hours with 25 μM or 50 μM of ML-60218, an inhibitor of RNA polymerase III [34]. Interestingly, a strong dose dependent decrease of ~60% to ~70–80% in *A3A* expression was observed respectively with 25 μM and 50 μM of ML-60218 respectively (Fig 5E). To confirm that ML-60218 treatment did not impair MeV replication in THP-1 cells, early (annexin V^+^ 7-AAD^−^ cells) and late apoptosis/necrosis (annexin V^+^ 7-AAD^+^) were analyzed. As visualized in S6A Fig, THP-1 cells treated with ML-60218 did not elicit apoptosis. In addition, SYBR RT-qPCR was performed on MeV *M* gene. As observed in S6B Fig, similar *M* RNA levels were detected in THP-1 ML-60218 treated cell lines, as well as in wild-type THP-1, indicating that the RNA Polymerase III inhibitor had no toxic effect on MeV replication.

**Fig 6:**
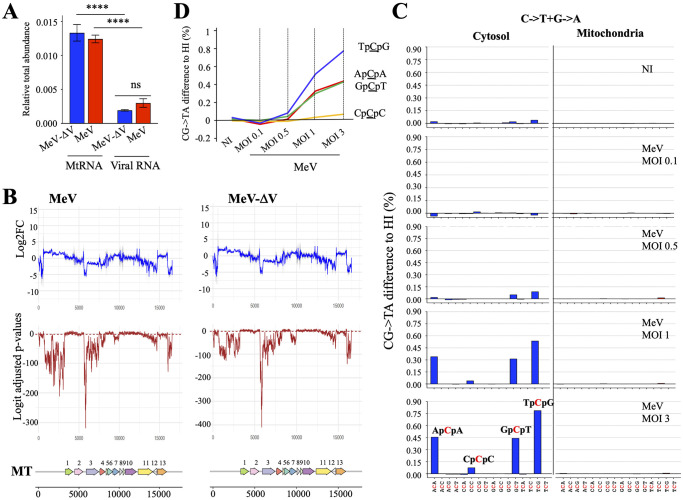
MtRNA binding to RIG-I, DNA mtDNA editing and context analysis. A) STrEP-tagged RIG-I obtained from total MeV or MeV-ΔV infected cell lysates were purified by affinity chromatography. Mitochondrial or viral RNA molecules biding to RIG-I was detected Next Generation Sequencing (NGS) analysis and was normalized to non-specific binding to the beads from STrEP-tagged-Cherry cells. Mean values and s.e.m. were calculated for three independent experiments in duplicate (n = 6), unpaired two-side Student’s t-test ***, p<0.005, ns: not statistically significant. B) Upon MeV and MeV-ΔV infections, the raw counts obtained for each position to calculate mtRNA binding efficiency and specificity between RIG-I and m-Cherry were performed using the DESeq2 package of R and were aligned to the mitochondrial genome. The grey area corresponds to the 95% confidence interval of the log2FC. The logit of the p-values was adjusted by the Benjamini-Hochberg method. The dashed line in red corresponds to the 5% threshold value. Bottom: Annotation of the mitochondrial genome. 1: MT-ND1; 2: MT-NT2; 3: MT-CO1; 4: MT-CO2; 5: MT-ATP8; 6: MT-ATP6; 7: MT-CO3; 8: MT-ND3; 9: MT-ND4L; 10: MT-ND4; 11: MT-ND5; 12: MT-ND6; 13: MT-CYB. C) Pattern of C->T and G->A mutations analyzed in mtDNA in the cytosol and mitochondria. Difference of mutation proportion between MeV at 0.1, 0.5, 1 and 3 MOI and NI sample. the different trinucleotides associated with the context are indicated at the bottom of the figure. The deaminated C is identified in red. D) C->T and G->A mutations occurring in specific contexts analyzed in cytosolic mtDNA.

To investigate the nucleic acid source of cytosolic RNA synthesis by DNA dependent RNA polymerase III that ultimately binds to RIG-I, human HEK293 cell line stably expressing One-STrEP-tagged-RIG-I protein were generated [28] and infected with MeV and MeV-ΔV. Cells overexpressing a STrEP-mCherry protein were used as control to evaluate the amount of RNA-nonspecific binding. At 24 hpi with-MeV or MeV-ΔV, RIG-I-specific RNA ligands were purified from total cell lysates by affinity chromatography. To identify the nature of bound RNA molecules to RIG-I, Next Generation Sequencing (NGS) analysis was performed using the Illumina technology and was normalized to non-specific RNA binding to STrEP-tagged-Cherry protein. We observed that there is ~6.5 times more mtRNA than viral RNA binding RIG-I, demonstrating that one of the main sources of RNA binding to RIG-I, eventually cascading into IFN/A3A productions, is of mitochondrial origin (Fig 6A).

To highlight the binding specificity of mtRNAs to RIG-I vs m-Cherry during MeV or MeV-ΔV infection, the raw sequence counts obtained for each position were processed using the DESeq2 R package and aligned to the mitochondrial genome. As observed in Fig 6B, the log2FC calculated between RIG-I and mCherry by the DESeq2 model (Fig 6B, blue curve) correlates with a significant p-value of transcripts bound to RIG-I (Fig 6B, red curve).

Altogether, these data reinforce that one of the important sources of RNA that bind to RIG-I, ultimately leading to IFN/A3A production during MeV infection, originates from the mitochondrial genome.

### Analyses of *MT-COI* mutations reveal an APOBEC3A signature

To analyze the mtDNA mutational spectrum, THP-1 infected cells at MOI 0.1, 0.5, 1 and 3 were subjected to digitonin fractionation to obtain purified mitochondria and cytosolic extracts (S6C Fig). To compare the mutation spectrum in both compartments, amplicons of the *MT-COI* region were generated by PCR from MeV infected cells and were analyzed by NGS (Fig 6C). Mutational contents in cytoplasmic and mitochondrial compartments were compared and analyzed for each sample based on substitutions within their respective contexts. As observed in Fig 6C, CG->TA substitutions were predominant in the cytosol and absent in the mitochondrial compartment. The other categories of mutations remained extremely low (not shown). In the cytosolic compartment, an increase in editing frequency was observed in a dose dependent manner in MeV infected cells (Fig 6C and 6D).

To prove that editing occurs outside of the mitochondria, A3A-A3H-V5 tagged constructs were transfected and mitochondrial localization was analyzed by confocal microscopy using TOM20 as a mitochondrial marker. As observed in S7 Fig, no colocalization of any A3 enzyme with the mitochondrial network was observed.

Altogether, these data demonstrated that upon mtDNA release in the cytosol, the IFN pathway was induced, leading to A3A production. In turn, A3A edited mtDNA in its specific 5′TpCpG signature to shut down the cytosolic DNA danger signal.

## Discussion

These findings highlight a novel mechanism by which MeV infection induces mitochondrial network alteration. This impairment is triggered by the MeV-V protein, which interacts with the mitochondrial enzyme ALAS1. Interactions between ALAS1 and MeV-V will then lead to the relocation of ALAS1 outside of the mitochondria, resulting in a shift in mitochondrial metabolism. Consequentially, damages to the mitochondrial network induced the release of mtDNA, leading to type I IFN and A3A production through the RNA polymerase III/RIG-I pathways.

To assess the effect of MeV and MeV-ΔV on cellular bioenergetics, an analysis of extracellular fluxes was performed using the Agilent Seahorse technology to evaluate the extracellular acidification rate (ECAR) and the oxygen consumption rate (OCR), which are representative indicators of lactic acid production and mitochondrial OXPHOS, respectively. As visualized in Fig 3C, we observed an enhanced mitochondrial activation exhibited by both MeV-ΔV and MeV. This phenomenon is not without precedent. Indeed, a comparable complexity of cellular metabolic alterations have also been reported for human cytomegalovirus [35,36] and rubella virus [37]. The increased mitochondrial metabolism could be a host cell response to the bioenergetic challenge induced by the virus biogenesis and its demand for cellular ATP. Upon infection, MeV appears to underline a more pronounced alteration of the mitochondrial network, demonstrating the implication of the MeV-V protein in the alteration of mitochondrial metabolism (Fig 3).

Our work also highlights the implication of MeV-V protein in mitochondrial network damages. Indeed, by using a two-hybrid screening, we demonstrated that MeV-V protein interacts with the mitochondrial enzyme ALAS1, and these data were validated by affinity purification (Fig 4). This interaction prevents the relocation of ALAS1 to the mitochondria and leads stepwise from alteration of the mitochondrial metabolism to the mitochondrial network damages (Fig 4). In the same vein, it has been observed, without describing a specific mechanism, that the influenza virus NS1A-binding protein (Ivns1abp) gene encoding the actin binding protein, Nd1 interacts with ALAS1 enzyme to inhibit the immune response [31]. A functional analysis of MeV-V cellular interaction network in infected cells identified by nano-LC-MS/MS analysis has been established, demonstrating that among the 245 human cellular proteins that copurified with MeV-V, 55 proteins belong to the mitochondrial network [30], indicating that different protein families belonging to the mitochondrial network can interact with MeV-V protein. Therefore, it is conceivable that the interaction of MeV-V protein with different mitochondrial proteins may dampen the interaction between ALAS1 and MeV-V. This could potentially involve an indirect effect of MeV-V on signaling pathways, leading to the disruption of the mitochondrial network.

Furthermore, we demonstrated the implication of cytosolic mtDNA in triggering the IFN signaling response. Indeed, it is now well reported that cytosolic DNA, whatever its origin, has an essential role in the IFN response, triggering the innate immunity signaling pathway. As observed in S5 Fig, THP-1 cell line stably overexpressing the cytoplasmic DNase I enzyme have shown that cytosolic DNA was the trigger for IFN/A3A signaling following MeV infection.

Several DNA sensors have been reported to play distinct functions in different cell types or in response to different viruses. Among them, cGAS can sense DNA viruses and trigger host immune responses upon infection [38,39]. Interestingly, it has recently been described that SARS-CoV-2, an RNA virus, could also be detected by cGAS [40]. Although this pathway may seem somewhat non-conventional for RNA viruses, cytosolic mtDNA released from hyperfused mitochondria during MeV infection could be captured by cGAS and trigger type I IFN induction [41].

We also observed that transcription of released cytosolic mtDNA by RNA polymerase III allowed for the synthesis of cytosolic mitochondrial RNA that binds RIG-I, leading to the activation of the IFN signaling pathway. A specific inhibitor used against RNA polymerase III (ML-60218) significantly reduced *A3A* expression by ~60% (Fig 5E), proving that this pathway was mainly followed by MeV, with an important source of RNA binding to RIG-I being of mitochondrial origin (Fig 6A and 6B).

As *A3A* is an ISG, this enzyme will be induced during IFN production through a paracrine or autocrine activation of the cell. Consequently, A3A relocation in the cytoplasm will lead to an increase of C->T editing of the cytosolic mtDNA. Indeed, by performing deep sequencing of cytosolic mtDNA, we observed, in a dose dependent manner, an increase of C->T editing occurring in a specific 5’TpCpG context, (Fig 6C and 6D), previously described as A3A’s specific signature [42,43]. Could editing of mtDNA be linked to autoimmune diseases? It has been shown that A3A induction may be correlated with autoimmune diseases such Systemic Lupus Erythematosus (SLE) [44]. Indeed, A3A-induced mtDNA editing could be involved in the pathophysiology of autoimmune diseases, by generating or increasing the autoantigen load, a key requisite for the progression of autoimmunity.

Altogether, these data reveal that the perturbation of mitochondrial structure and function upon MeV infection can alter the innate immune responses. The most direct effect of mitochondria on immune response is consequently due to mitochondrial damage and disruption of mitochondrial metabolism *via* the interaction of MeV-V with ALAS1 enzyme, which aggravates the physiological mitochondrial dynamics and functions. Since many of the viral proteins that interacts with mitochondrial proteins are essential for viral replication *in vivo*, these proteins might serve as critical drug targets for generating therapeutics against viral infections.

## Materials and methods

### Ethics statement

All animal experiments were performed according to French legislation in compliance with the European Communities Council Directives (2010/63/UE, French Law 2013–118, February 6, 2013) and according to the regulations of Institut Pasteur Animal Care Committees. The Animal Experimentation Ethics Committee (CETEA 89) of the Institut Pasteur approved this study (dap210133) before experiments were initiated. The animals were manipulated in class III safety cabinets in the Institut Pasteur animal facilities accredited by the French Ministry of Agriculture for performing experiments on live rodents.

### Reagents

RNA polymerase III inhibitor (ML-60218) was from Merck Millipore. MDA5 (D74E4, rabbit mAb #5321), STING (antibody #3337), phospho-IRF-3 (Ser396) (4D4G, rabbit mAb #4947), IRF-3 (D6I4C, XP rabbit mAb #11904), RIG-I (D14G6, rabbit mAb #3743), MAVS (antibody #3993), TBK1/NAK (D1B4, rabbit mAb #3504), phospho-TBK1/NAK (Ser 172) (D52C2, rabbit mAb, #5483), IKKε (D20G4, rabbit mAb, #2905), cGAS (E9G9G, rabbit mAb, #83623), beta actin HRP conjugate (13E5, rabbit mAb # 5125), anti-rabbit IgG HRP-linked (antibody #7074), histone H2AX (antibody #2595), antibodies were from Cell Signaling Technology. GAPDH (mouse mAb, #G8795) was from Sigma Aldrich. Anti-mouse IgG HRP-linked (antibody #NA931V) was from GE Healthcare. Monoclonal anti-β-actin-peroxidase (antibody #A3854) and digitonin (D141) were from Sigma Aldrich. Anti-rabbit MEK1/2 (#8727), anti-rabbit AIF (#5318) and anti-rabbit Histone H3 (#4499) were from cell signaling, anti-rabbit ALAS1 (#MA5-35584) were from Invitrogen. TURBO DNase (2U/μl, #AM2238) was from Invitrogen. GlycoBlue Coprecipitant (AM9515) was from Applied Biosystems. TOM20 (EPR15581-39, rabbit mAb, #ab186734) and mCherry (1C51, rabbit mAb, #ab125096) were from Abcam. RIG-I (D33H10, rabbit mAb, #4200) and mouse anti-rabbit IgG (conformation specific, L27A9, mAb #3678), cell lysis buffer (#9803), protein A magnetic beads (#73778), glycine (#7005) and PMSF (#8553) were from Cell Signaling. Recombinant RNasin Ribonuclease inhibitor (#N2515) was from Promega. Secondary Alexa Fluor 488 goat anti-mouse (#A11029), Alexa Fluor 488 goat anti-rabbit (#A11034), Alexa Fluor 633 goat anti-mouse (#A21053), Alexa Fluor 647 goat anti-rabbit (#A21244), Fluoromount-G with DAPI (#00-4959-52) and SlowFade Diamond Antifade Mountant without DAPI (#S36972) were from Invitrogen. DAPI solution 1.0 mg/ml (564907) was from BD Pharmingen. Monoclonal Alexa Fluor 647 Mouse anti-H2AX (pS139) (#560447) was from BD Pharmingen. Annexin V Apoptosis Detection kit (888007) with Annexin V efluor 450 (48-8006-69) and Fixable viability Dye eFluor 780 (65-0865-14) were from eBioscience. JetPRIME (#114–07) was from Polyplus Transfection and Lipofectamine RNAiMAX (#13778075) was from Invitrogen. NucleoSpin Gel and PCR Clean-up kit (#740609) was from Macherey-Nagel. Quant-iT dsDNA Assay kit, High sensibility (#Q33120) was from Invitrogen and NEBNext DNA Library Prep kit (#E7645) and NEBNext Multiplex Oligos for Illumina (#E7600) were from New England BioLabs.

### Cell culture

THP-1 cells (ATCC TIB-202) were maintained in RPMI-1640 (Gibco), supplemented with 50 U/ml penicillin and 50 μg/ml streptomycin, 10% heat-inactivated fetal calf serum, and 0.00035% β-mercaptoethanol. The HEK-293T and HeLa cells, were maintained in DMEM (Gibco) supplemented with 50 U/ml penicillin and 50 μg/ml streptomycin, 10% heat-inactivated fetal calf serum. Vero (African green monkey kidney cells) cells were maintained in DMEM (Gibco) supplemented with 10% heat-inactivated fetal calf serum and 100 U/ml of penicillin-streptomycin. Cells were grown at 37°C in a humidified atmosphere containing 5% CO_2_.

### Establishment of THP-1 DNase I cell lines

Human wild type and catalytic mutant (N7A) DNase I was inserted in a pTRIP lentiviral vector under the EF1α promoter. 4.10^6^ HEK-293T were seeded in 10 cm petri dishes in 8 mL complete medium and transfected 24 hours later with 1.5μg of pTRIP vector, 1μg of p.8.74 plasmid (encoding HIV gag and pol) and 1μg VSV G plasmid (encoding the VSV envelop) using Fugene HD (Promega) transfection reagent. Culture supernatant was collected 24 hours post transfection and filtered (0.45μm). 0.6.10^6^ THP-1 cells were seeded in 6-well plate in 1mL complete medium and exposed 24h later to 2mL of filtered supernatant. mCherry positive cells were then selected twice by FACS to establish stable cell lines.

### Measles virus infection

The Schwarz vaccine strain MeV and MeV-ΔV were previously described [28]. Briefly, V protein expression from MeV was knock-down following a two-step PCR strategy to generate MeV-ΔV virus. These PCRs introduced a mutation interfering with RNA editing (the native sequence UUAA**A**AAGGGCACAGA was mutated to UUAA**G**AAGGGCACAGA) [28]. MeV with a GFP (MeV-GFP) was previously described [27] while MeV-ΔV-GFP was performed by cloning the *GFP* ORF via Sbfi and Tth111I sites from PTM3-egfp plasmid and put the fragment in the MeV-ΔV virus. Unless overwise specified, 10^6^ THP-1 cells were seeded in 12-well plates for infection in 500μL non-supplemented RPMI. 500μL 2X supplemented RPMI was added 2 hours post-infection to restore the appropriate concentration of supplements. THP-1 cells were infected with MeV at MOI 0.1, 0.5, 1 or 3 for 24 hours. As negative control, cells were infected at a MOI 0.5 with MeV, previously inactivated at 70°C for 30 minutes. For both infections, viruses were absorbed in serum-free RPMI for a defined period at 37°C before dilution with complete RPMI. To study the role of the RNA polymerase III, inhibitor was added to the culture medium at final concentrations of 25 or 50 μM for ML-60218 (Merck Millipore) 2 hpi. Monolayers of Vero or THP-1 cells were infected with MeV, MeV-ΔV, MeV-GFP and MeV-ΔV-GFP at MOI 0.1. At various times post infection, cells were scraped into culture medium and froze at -80°C. After medium clarification of cell debris, virus titers were determined. For this purpose, Vero cells were seeded into 96-well plates (7,500 cells/well) and infected with serial 1:10 dilutions of virus sample in DMEM-5% FCS. After incubation for 7 days, cells were stained with crystal violet, and the TCID_50_ values were calculated by using the Karber method.

One-STrEP Tag Purification-HEK-293T (8.10^7^) cells were either infected with rMeV/STrEP-V or with the negative control rMeV/STrEP-Cherry viruses at MOI 1. At 24 hpi, cells were lysed and tagged protein co-complexes were purified and analyzed by Western Blot as previously described [30].

### Mice infection with MeV and MeV-ΔV

Mice deficient for IFNα/β receptors and expressing human MeV receptor CD46 (IFNAR^-/-^hCD46Tg) were produced and housed under pathogen-free conditions at the Institut Pasteur animal facility. Groups of 6 to 8-week-old mice were intraperitoneally injected with 10^5^ TCID_50_ of either MeV [27] or MeV-ΔV [28] or the control of 100μl PBS. 200μl whole blood was collected before infection (day -24 hours). At 24 hpi, mice were sacrificed, spleen samples were collected, and 200μl whole blood was stored in RNA protect tubes (Qiagen). Spleens were manually homogenized and passed through 30μM filters as the red blood cells were lysed. CD11b^+^ cells were isolated using magnetic bead coupled antibodies according to the manufacturer’s recommendations (Miltenyi Biotech). CD11b^+^ cells were either kept intact, or further processed into their cytosolic and mitochondrial fractions as described above. Whole blood was heat-inactivated for 1 hour at 56°C, following either DNA or RNA extraction by immunoprecipitation methods or using RNA Plus Mini kit (Qiagen), respectively.

### Quantitative PCR

Total RNA was extracted using the RNeasy Plus Mini kit (Qiagen) and synthesis of cDNA was performed with 1 μg RNA using the Quantitect Reverse Transcription kit (Qiagen) according to the manufacturer’s instructions. Total DNA was extracted using Master Pure Complete DNA and RNA extraction kit according to the manufacturer’s instructions. Quantitative PCR was performed on cDNA and DNA samples with TaqMan Fast Advanced Master Mix (Applied Biosystems). Expression of *APOBEC3s*, *IFN-α* and *IFN-β* genes were essayed using Real-Time PCR on TaqMan (Applied Biosystems) along with *RPL13A* and *HPRT1* as reference genes. Primers for the amplification of *A3* as well as for the quantification of mtDNA (*MT-COI*) were previously described [45,46]. For MeV and MeV-ΔV RNA quantification, total RNA of infected cells was extracted by using the Nucleo Spin DxVirus kit (Macherey Nagel Bioanalysis). Reactions were performed with 400 ng of total RNA with the TaqMan RNA-to-Ct 1-Step kit (Thermo Fisher Scientific) for one-step RT-qPCR analyses. Reactions were performed in a final volume of 20 μl. The full-length MeV genome was amplified with *MeV-H-L* fwd and *MeV-H-L* rev primers with the specific TaqMan *MeV-H-L* probe chosen in the *H-L* intergenic region of the MeV genome. *β-actin* mRNA was amplified as housekeeping gene with *β-actin* fwd and *β-actin* rev primers in with the specific TaqMan *β-actin* probe. Primers, probes and PCR conditions can be found in S1 Table.

### Western blotting

Cells were treated with lysis buffer (0.5M Tris-HCl, pH 7.4, 1.5M NaCl, 2.5% deoxycholic acid, 10% NP-40, 10mM EDTA and EDTA-free Protease Inhibitor [Millipore]) followed by sonication and centrifugation at 20,000 x g for 20 minutes. Clarified lysates were loaded on NuPAGE 4 to 12% bis-Tris gels (Invitrogen) and transferred to a nitrocellulose membrane (Invitrogen) according to the manufacturer’s instructions. Primary antibodies were incubated overnight in 5% BSA, followed by an HRP-linked secondary antibodies (1/5,000) incubation for 1 hour. Membranes were revealed using a chemiluminescence assay (Pierce). β-Actin HRP antibody (1/50,000) was used as a loading control. Purity of cellular fractionation was confirmed using anti-MEK1/2 as a cytosolic protein marker, anti-AIF as a mitochondrial marker and anti-γ-H2AX as a nuclear protein marker (1/1000). Anti-ALAS1 (1/1000) and the cellular fractionation validation was performed on whole cell lysates as well as mitochondrial and cytosolic partitions. 18 h post-MeV infection, cell lysates were assayed for proteins of the IFN pathway using antibodies from the RIG-I pathway antibody kit (Cell Signaling) according to manufacturer recommendations. Anti-MeV-V rabbit polyclonal antibody was kindly provided by Dr. Kaoru Takeuchi [47]. DNase I was assayed using an anti-DNase I antibody (1/500; Thermo Scientific). After imaging, proteins were quantified by normalization to β-actin or MEK1/2 using ImageJ software.

### Immunofluorescence

For DNase I localisation visualization, HeLa cells were plated in Nunc Lab-Tek II chamber slides (Thermofisher) and transfected with 1μg of pcDNA 3.1 DNase I-mCherry plasmid using Fugene HD (Promega) transfection reagent. 48 hours post-transfection, cells were washed with PBS and fixed with 4% paraformaldehyde for 15 minutes and permeabilized with 0.1% triton X-100 for 10 minutes. Cells were then washed with PBS before and Vectashield mounting media (VectorLabs) containing nuclei stain DAPI (1 μg/mL; BD Biosciences) was used for all slide preparation and imaging was performed with a Leica SP5 confocal microscope. For mitochondrial network visualization, THP-1 cells were plated in μ-Slide chamber coverslips (Ibidi) for MeV infection. 24 hpi, cells were washed and fixed and permeabilized as described above. Cells were then washed with PBS before primary antibody incubation of rabbit monoclonal anti-TOM20 antibody (1/250; Abcam) in PBS-bovine serum albumin (BSA; 0.5%) for 1 h at room temperature. Secondary antibody was Alexa Fluor 647 goat anti-rabbit (1/750; Invitrogen) for 45 min at room temperature concurrently with Alexa Fluor 555 Phalloidin. Vectashield mounting media (VectorLabs) containing nuclei stain DAPI (1 μg/mL; BD Biosciences) was used for all slide preparation and imaging was performed with a Leica SP8 confocal microscope. For APOBEC3 localization visualization, HeLa cells were plated in μ-Slide chamber coverslips (Ibidi) and transfected with 400ng APOBEC3 plasmids for 24 hours. Cells were washed and fixed and permeabilized as described above. A primary mouse anti-V5 antibody (1/200; Invitrogen) was used for APOBEC3 staining and TOM20 was used for mitochondria. Secondary antibodies were anti-mouse Alexa Fluor 488 (1/1000; Invitrogen), anti-rabbit Alexa Fluor 647 (1/1000; Invitrogen) concurrently with Alexa Fluor 555 Phalloidin. Vectashield mounting media (VectorLabs) containing nuclei stain DAPI (1 μg/mL; BD Biosciences) was used for all slide preparation and imaging was performed with a Leica SP8 confocal microscope.

### Subcellular fractionation

5.10^6^ cells were collected 24 hpi, washed in PBS and split: 10^6^ cells were kept for Western Blot on whole cell extract (WCE), 2.10^6^ cells were kept for total DNA extraction and 2.10^6^ cells were lysed in 0.5mL digitonin buffer (150 mM NaCl, 50 mM Hepes (pH 7.4), 0.03mM, protease and phosphatase inhibitors cocktail) for 10 minutes at 4°C. After centrifugation (2000g, 10 minutes, 4°C) the supernatant was centrifuged three times (20000g, 20 minutes, 4°C), the last supernatant constitutes the cytosolic fraction and was split in two, one half for DNA extraction, the other for western blotting. The initial pellet was resuspended in 300μL NP-40 buffer (150 mM NaCl, 50 mM HEPES pH7.4, 1% NP-40, protease and phosphatase inhibitors cocktail), for 30 minutes at 4°C. After centrifugation (7000g, 10 minutes, 4°C) the pellet and supernatant were split in half for western blotting and DNA extraction, the pellet constitutes the nuclear fraction and the supernatant the crude mitochondrial lysate.

### Extraction and characterization of RIG-I bound ligands

Briefly, infected HEK-293 cells stably expressing One-STrEP-tagged RIG-I or Cherry protein were lysed with MOPS lysis buffer (20 mM MOPS-KOH pH7.4, 120 mM of KCl, 0.5% Igepal, 2 mM ß-Mercaptoethanol), supplemented with 200 U/mL RNasin (Promega) and Complete Protease Inhibitor Cocktail (Roche). An aliquot of each cell lysate was used to perform total RNA purification using TRI Reagent LS (Sigma). The remaining cell lysate was kept to purify the RIG-I/RNA, or Cherry/RNA complexes by affinity chromatography using StrepTactin Sepharose High Performance beads (GE Healthcare). RNAs that had co-precipitated with either RIG-I or Cherry protein were isolated using TRI Reagent LS. RNA was dissolved in DNase-free and RNase-free ultrapure water. Extracted RNAs were analyzed using the Nanovue (GE Healthcare) and Bioanalyser RNA Nano kit (Agilent). 300 ng of each RNA sample was treated for library preparation using the TruSeq Stranded mRNA Sample Preparation kit (Illumina). Sequencing was performed on the Illumina Hiseq2000 platform to generate single-end 51 bp reads bearing strand specificity [28]. Three biological experiments were performed.

For the analysis of total and RIG-I/Cherry-specific RNAseq, reads were aligned against Human Genome Reference (GRCh37) with Bowtie2 v2.3.5.1 [48]. Reads without a positive match against GRCh37, were aligned against the Measles reference genome (NC_001498). Resulting SAM files were analyzed with Samtools v1.13 [49]. The statistical analyses were performed with SHAMAN (shaman.pasteur.fr) [50]. The count of reads aligned against Human genome chromosomes and Measles virus were normalized by using the weighted non-null normalization method described in [50]. Resulting P values were adjusted according to the Benjamini and Hochberg procedure.

### Deep sequencing

For the analysis of A3-induced mutations in mitochondrial DNA, cells were infected with MeV at MOI 0.1, 0.5, 1 and 3 and were compared to non-infected cells (NI) for 24 hours. Total DNA from cytosolic fractions was extracted and *MT-COI* gene was amplified by standard PCR using the primers dHCoxI fwd and dHCoxI rev (S1 Table). For *MT-COI* gene, conditions were 5 min at 95°C, then 35 cycles of 30 sec at 95°C, 30 sec at 60°C and 1 min at 72°C, followed by 10 min at 72°C. PCR products were purified with the NucleoSpin Gel and PCR Clean-up kit (Macherey-Nagel) and quantified using the Quant-iT dsDNA Assay kit, High sensibility (Invitrogen) according to the manufacturer’s instructions. The preparation of the DNA library was performed with the NEBNext DNA Library Prep kit and NEBNext Multiplex Oligos for Illumina (New England BioLabs) for the fragmentation and multiplex adapters ligation steps. Deep sequencing was performed with Illumina cBot and GAIIX technology.

Sequenced reads were cleaned of library adapter and base pairs occurring at 5′ and 3′ ends with a Phred quality score <20 were trimmed off by Alientrimmer [51] (https://gitlab.pasteur.fr/GIPhy/AlienTrimmer). Reads with a minimum length of 35 were subject for the next analysis.

For the SNP analysis, duplicated reads were removed with Picard-tools v2.23.3 (https://broadinstitute.github.io/picard/; Broad Institute). Single Nucleotide Variants (SNP) detection was performed with bcftools mpileup V1.13 [49] to extract and detect their occurrence. This workflow of analysis was resumed in a nextflow workflow [52] available at https://gitlab.pasteur.fr/aghozlan/measles.

### Mitochondria behavior analysis

All data intensities were normalized using the min-max intensity. The TOM20 signal labeling the mitochondria is deconvoluted using the Huygens method (SVI, Huygens professional. 19.04). Cells were segmented using the DAPI and Alexa 555 signals, first by denoising the DAPI channel with a combination of a median filter of radius = 6 and a Gaussian filter of sigma = 3, followed by an Otsu threshold [53] and a labeling filter. A similar approach is applied on the Alexa555 signal, with a median filter of radius = 6 and a Gaussian filter of sigma = 3, followed by a mean threshold and a marker-based watershed using the segmented nucleus labels as seeds. Finally, small holes and objects are removed using morpho-mathematical filters. The deconvoluted mitochondrial signal was segmented by applying an Otsu threshold [53] followed by a labeling filter, and were associated with their respective cells. We then computed for each cell the total volume of mitochondria.

## Supporting information

S1 Fig Western blot and mitochondrial network analyses. A) Western blot analysis of the dsDNA breaks with anti-γ-H2AX antibodies at 24 hpi with MeV at different MOIs. B) Western blot analysis of the pro-apoptotic Bax and Bak protein expressions in THP-1 cells, upon MeV infection at MOI 1 and 3. β-actin protein expression was used as loading control. kDa: kilo Dalton. NI: non-infected cells; HI: infected cells with heat inactivated virus. C) Mitochondrial segmentations were deconvoluted by applying an Otsu threshold followed by a labeling filter and associated with their respective cells. A raw quantification is calculated for each cell with the total volume of mitochondria, the number of mitochondria, and the sphericity of each mitochondria.
(TIFF)

S2 Fig Growth kinetics of Measles viruses in Vero and THP-1 cell lines. A-B) Growth kinetics of MeV and MeV-ΔV in Vero and THP-1 cells. C-D) Growth kinetics of MeV-GFP and MeV-ΔV-GFP in Vero and THP-1 cells. For the different Measles viruses used, relative quantification of MeV genome (detection of *H-L* intergenic region) was performed at each time-point: 12, 24, 36, 48, 60, 72h by RT-qPCR [54]. Virus titers (TCID_50_/ml) were characterized by limiting dilution. Mean values and s.e.m. were calculated for two independent experiments in duplicate.
(TIFF)

S3 Fig Analysis of mitochondrial activity by the Seahorse extracellular flux analyzer. A) Measure of the oxygen consumption rate (OCR, pMoles/min), indicative of OXPHOS in Vero cells infected with MeV or MeV-ΔV viruses. After establishing a baseline, oligomycin (2 μM), FCCP (0.8 μM), and rotenone (0.5 μM) were sequentially added. B) The OCR was measured at 24 hpi under basal and infected conditions using Seahorse technology with a Mito stress test kit. Sequential injection of oligomycin (Oligom.), FCCP, and rotenone/antimycin A (Rot./Ant.) is indicated, mean values and s.e.m. were calculated for three independent experiments in duplicate (n = 6). C) The spare respiratory capacity analysis in NI and infected conditions. Data from were subjected to two-way ANOVA, followed by a Sidak post hoc test, *, p < 0.05, **, p < 0.01, <****, p < 0.001. D) Profiling of ECAR (mpH/min) in control and infected cells was measured in the same experiments as described in A and B, mean values and s.e.m. were calculated for three independent experiments in duplicate (n = 6).
(TIFF)

S4 Fig DNase I overexpression reduced *A3A* upregulation upon MeV infection. A) Yeast two-hybrid system form mapping MeV protein interactions. B) Stable DNase I or a catalytically inactive mutant DNase I_N7A_ overexpressed THP-1 cell lines were generated. Western blot analysis of the DNase I or DNase I_N7A_ protein expression in THP-1 cells, transfected or not with the DNase I-mCherry plasmid. β-actin protein expression was used as loading control. kDa: kilo Dalton. C) Cellular localization of the DNase I or DNase I_N7A_ proteins by immunofluorescence after transfection of constructions in HeLa cells. Nuclei were stained using DAPI (blue) and mCherry constructions were shown in red. ep: empty plasmid. D) Flow cytometry analysis of early apoptosis (annexin V^+^ 7-AAD^−^ cells) and late apoptosis/necrosis (annexin V^+^ 7-AAD^+^ cells) at 24h post-transfection in HeLa cells. Positive control was performed by incubating actinomycin. Mean values an s.e.m. were calculated for three independent experiments in duplicate (n = 6), unpaired two-side Student’s t-test, ***, p<0.005, ns: not statistically significant.
(TIFF)

S5 Fig Relative *APOBEC3A* and MeV *M* mRNA quantification upon MeV infections in cells stably expressing DNase I. A) *APOBEC3A* profiling by RT-qPCR at 24 hours by incubating THP-1 with 20 μg/ml of PGN. B) *APOBEC3A* profiling by RT-qPCR at 24h post-tranfection of 250 ng of DNA. C) *APOBEC3A* profiling by RT-qPCR at 24 hpi with MeV at MOI 1 infection in THP-1 expressing DNase I or not, negative control was performed with a heat inactivated (HI) virus. Control was performed. Data were normalized to the *RPL13A* housekeeping gene. D) MeV *M* RNA profiling by RT-qPCR at 24 hpi in stable DNase I or a catalytically inactive mutant DNase I_N7A_ overexpressed THP-1 cell lines. A-D) Mean values and s.e.m. were calculated for three independent experiments in duplicate (n = 6), unpaired two-side Student’s t-test, ***, p < 0.005 and ns: not statistically significant.
(TIFF)

S6 Fig MeV *M* mRNA quantification in cells treated with an inhibitor of RNA polymerase III. A) Flow cytometry analysis of early apoptosis (annexin V^+^ 7-AAD^−^ cells) and late apoptosis/necrosis (annexin V^+^ 7-AAD^+^ cells) in HeLa cells incubated with 25μM and 50μM of ML-60218. Positive control was performed by incubating with Etoposide. The error bars represent the s.d. from three independent experiments using a two-way analysis of variance (ANOVA test). B) MeV *M* mRNA profiling by RT-qPCR at 24 hpi in THP-1 treated with 25μM or 50μM of ML-60218, negative control with a heat inactivated (HI) virus. Data were normalized to the *RPL13A* housekeeping gene. Mean values and s.e.m. were calculated for three independent experiments in duplicate (n = 6), unpaired two-side Student’s t-test, ***, p < 0.005 and ns: not statistically significant, nd: not determined. C) Purity control of cellular fractions by immunoblotting, using β-actin to indicate the cytosol, AIF (apoptosis inducing factor) for mitochondria and MEK1/2 as a marker of cytosolic proteins. kDa: kilo Dalton.
(TIFF)

S7 Fig Cellular localization of APOBEC3 proteins. Confocal microscopy of V5-tagged A3A-A3H plasmids performed in HeLa cells at 24 hours post transfection. Nuclei are stained using DAPI (blue), and mitochondrial network detection was performed with anti-TOM20 (red), APOBEC3 with anti-V5 (green), and actin was visualized using phalloidin (yellow). ep: empty plasmid.
(TIFF)

S1 Table Compendium of primers used for PCR, qPCR and SYBR green. fwd: forward, rev: reverse.
(DOCX)

S1 Movie 3D representation of NI cells realized with IMARIS 9.9.1. (MP4)

S2 Movie 3D representation of MeV-ΔV infected cells realized with IMARIS 9.9.1. (MP4)

S3 Movie 3D representation of MeV infected cells realized with IMARIS 9.9.1. (MP4)
