# Supplementary figures and images for: Antagonism of ALAS1 by the Measles Virus V protein contributes to degradation of the mitochondrial network and promotes interferon response
Source: PLoS Pathog. 2023 Feb 21;19(2):e1011170. doi: 10.1371/journal.ppat.1011170 (PMC9983871; doi:10.1371/journal.ppat.1011170)

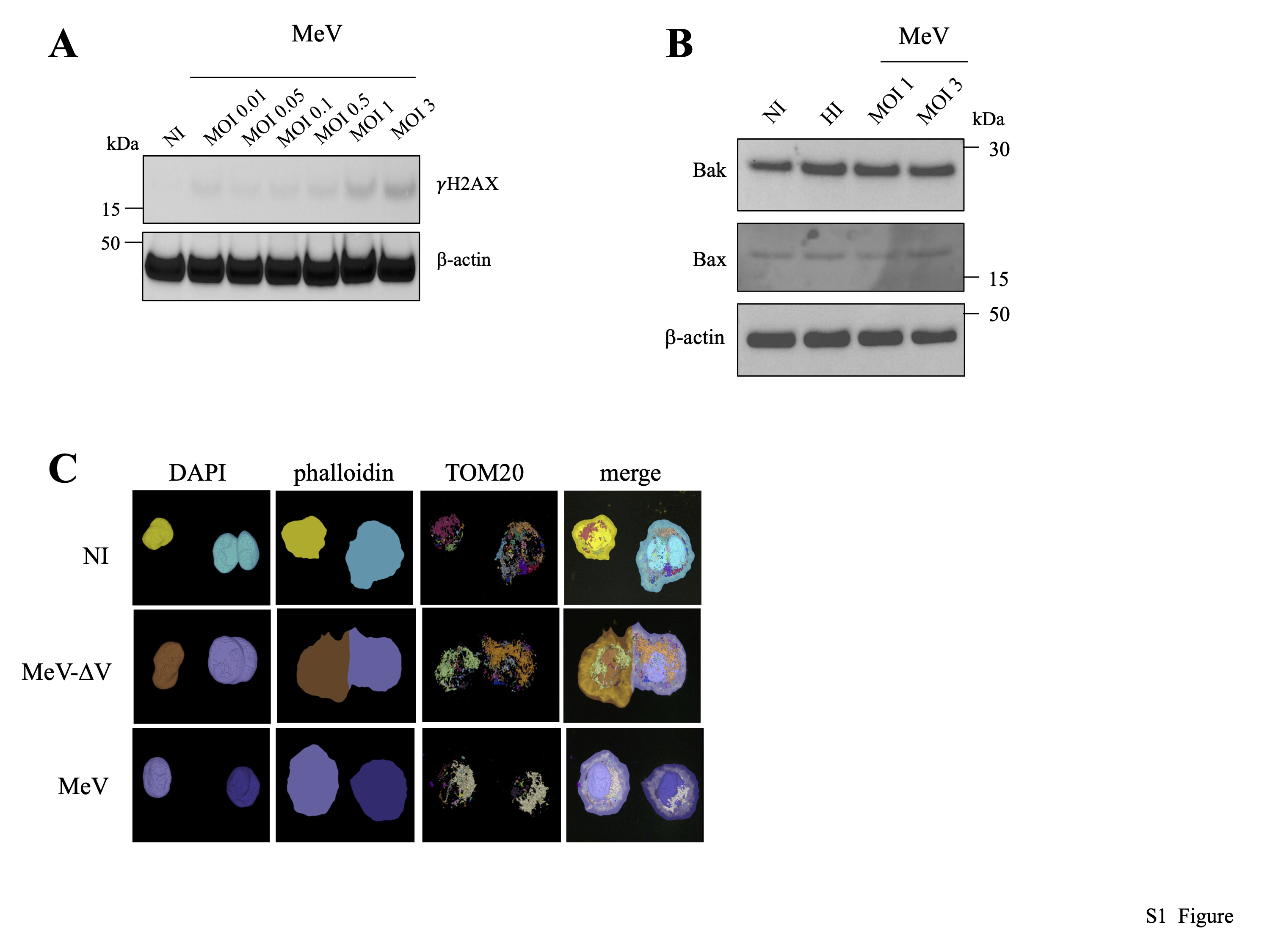

Supplement: S1 Fig — A) Western blot analysis of the dsDNA breaks with anti-γ-H2AX antibodies at 24 hpi with MeV at different MOIs. B) Western blot analysis of the pro-apoptotic Bax and Bak protein expressions in THP-1 cells, upon MeV infection at MOI 1 and 3. β-actin protein expression was used as loading control. kDa: kilo Dalton. NI: non-infected cells; HI: infected cells with heat inactivated virus. C) Mitochondrial segmentations were deconvoluted by applying an Otsu threshold followed by a labeling filter and associated with their respective cells. A raw quantification is calculated for each cell with the total volume of mitochondria, the number of mitochondria, and the sphericity of each mitochondria. (TIFF) [file ppat.1011170.s001.tiff]

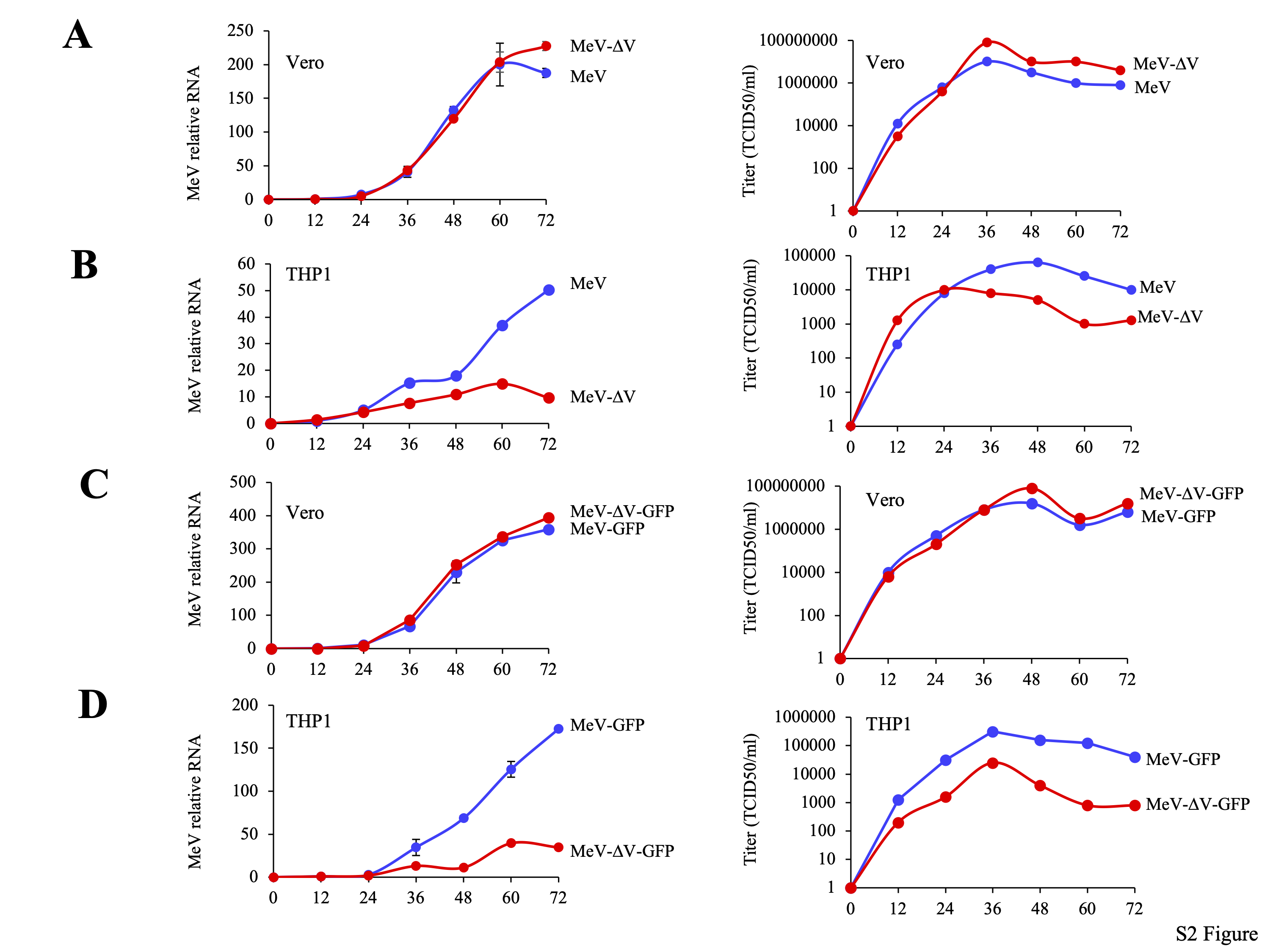

Supplement: S2 Fig — A-B) Growth kinetics of MeV and MeV-ΔV in Vero and THP-1 cells. C-D) Growth kinetics of MeV-GFP and MeV-ΔV-GFP in Vero and THP-1 cells. For the different Measles viruses used, relative quantification of MeV genome (detection of H-L intergenic region) was performed at each time-point: 12, 24, 36, 48, 60, 72h by RT-qPCR [54]. Virus titers (TCID50/ml) were characterized by limiting dilution. Mean values and s.e.m. were calculated for two independent experiments in duplicate. (TIFF) [file ppat.1011170.s002.tiff]

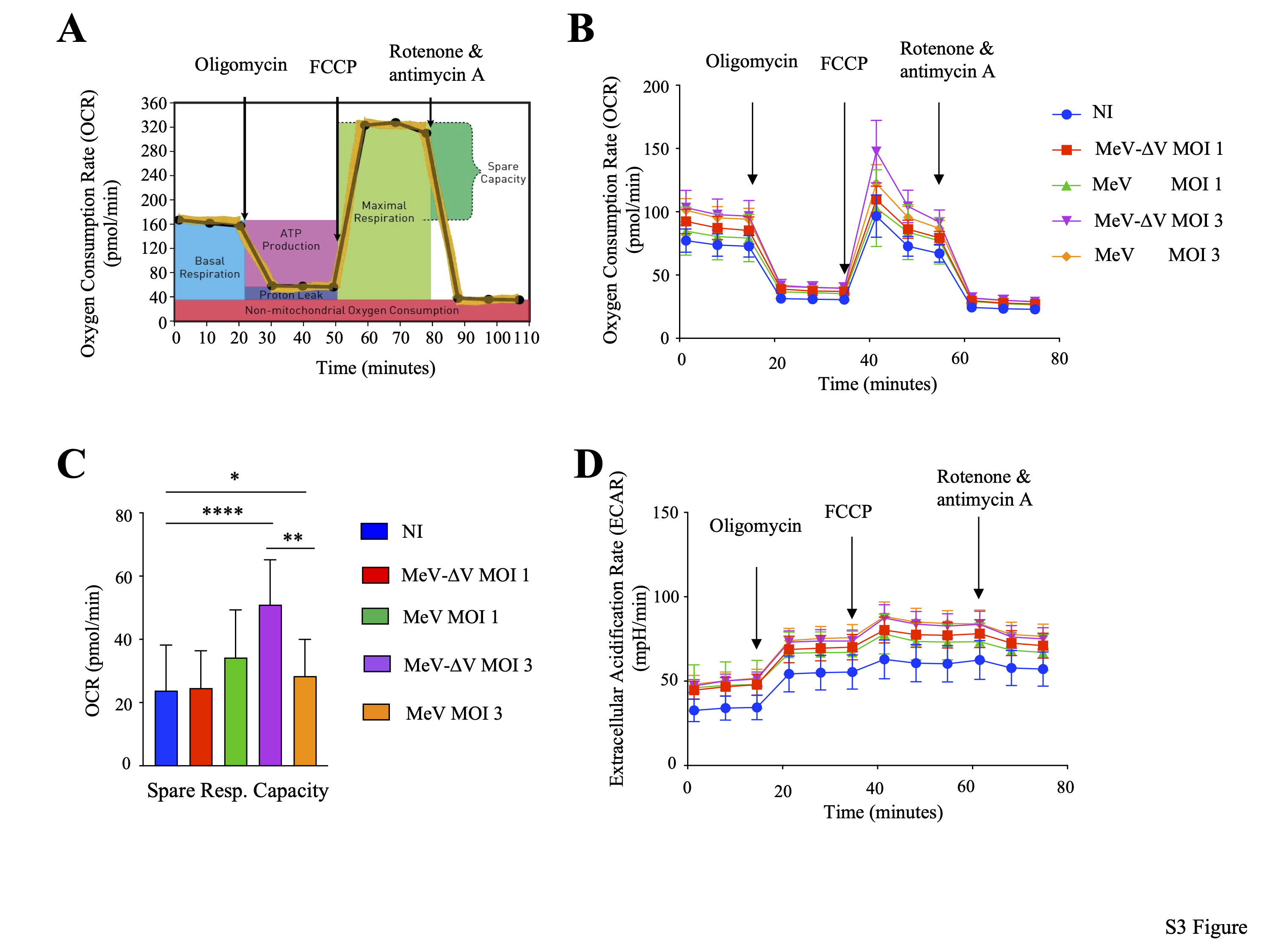

Supplement: S3 Fig — A) Measure of the oxygen consumption rate (OCR, pMoles/min), indicative of OXPHOS in Vero cells infected with MeV or MeV-ΔV viruses. After establishing a baseline, oligomycin (2 μM), FCCP (0.8 μM), and rotenone (0.5 μM) were sequentially added. B) The OCR was measured at 24 hpi under basal and infected conditions using Seahorse technology with a Mito stress test kit. Sequential injection of oligomycin (Oligom.), FCCP, and rotenone/antimycin A (Rot./Ant.) is indicated, mean values and s.e.m. were calculated for three independent experiments in duplicate (n = 6). C) The spare respiratory capacity analysis in NI and infected conditions. Data from were subjected to two-way ANOVA, followed by a Sidak post hoc test, *, p < 0.05, **, p < 0.01, <****, p < 0.001. D) Profiling of ECAR (mpH/min) in control and infected cells was measured in the same experiments as described in A and B, mean values and s.e.m. were calculated for three independent experiments in duplicate (n = 6). (TIFF) [file ppat.1011170.s003.tiff]

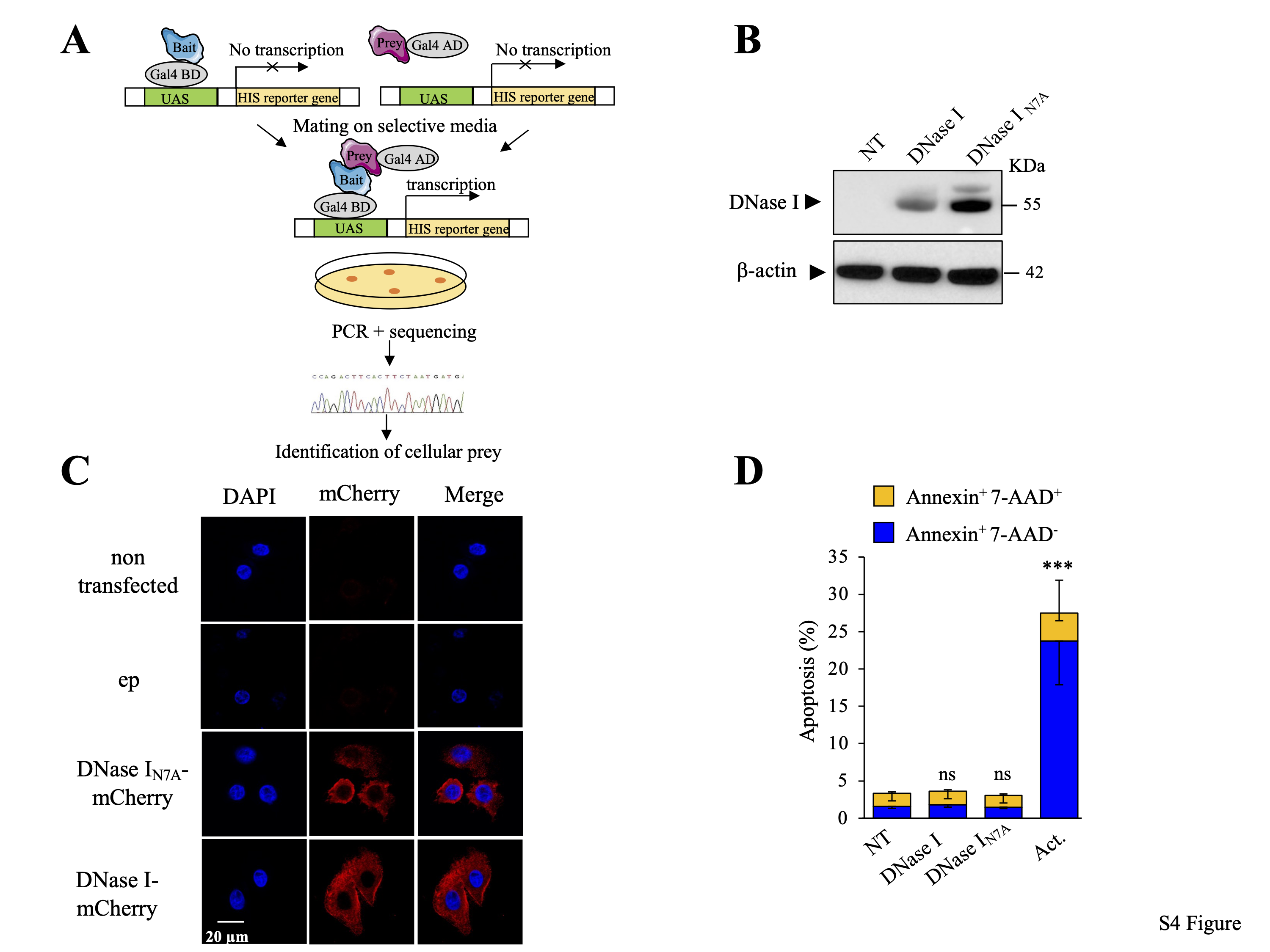

Supplement: S4 Fig — A) Yeast two-hybrid system form mapping MeV protein interactions. B) Stable DNase I or a catalytically inactive mutant DNase IN7A overexpressed THP-1 cell lines were generated. Western blot analysis of the DNase I or DNase IN7A protein expression in THP-1 cells, transfected or not with the DNase I-mCherry plasmid. β-actin protein expression was used as loading control. kDa: kilo Dalton. C) Cellular localization of the DNase I or DNase IN7A proteins by immunofluorescence after transfection of constructions in HeLa cells. Nuclei were stained using DAPI (blue) and mCherry constructions were shown in red. ep: empty plasmid. D) Flow cytometry analysis of early apoptosis (annexin V+ 7-AAD− cells) and late apoptosis/necrosis (annexin V+ 7-AAD+ cells) at 24h post-transfection in HeLa cells. Positive control was performed by incubating actinomycin. Mean values an s.e.m. were calculated for three independent experiments in duplicate (n = 6), unpaired two-side Student’s t-test, ***, p<0.005, ns: not statistically significant. (TIFF) [file ppat.1011170.s004.tiff]

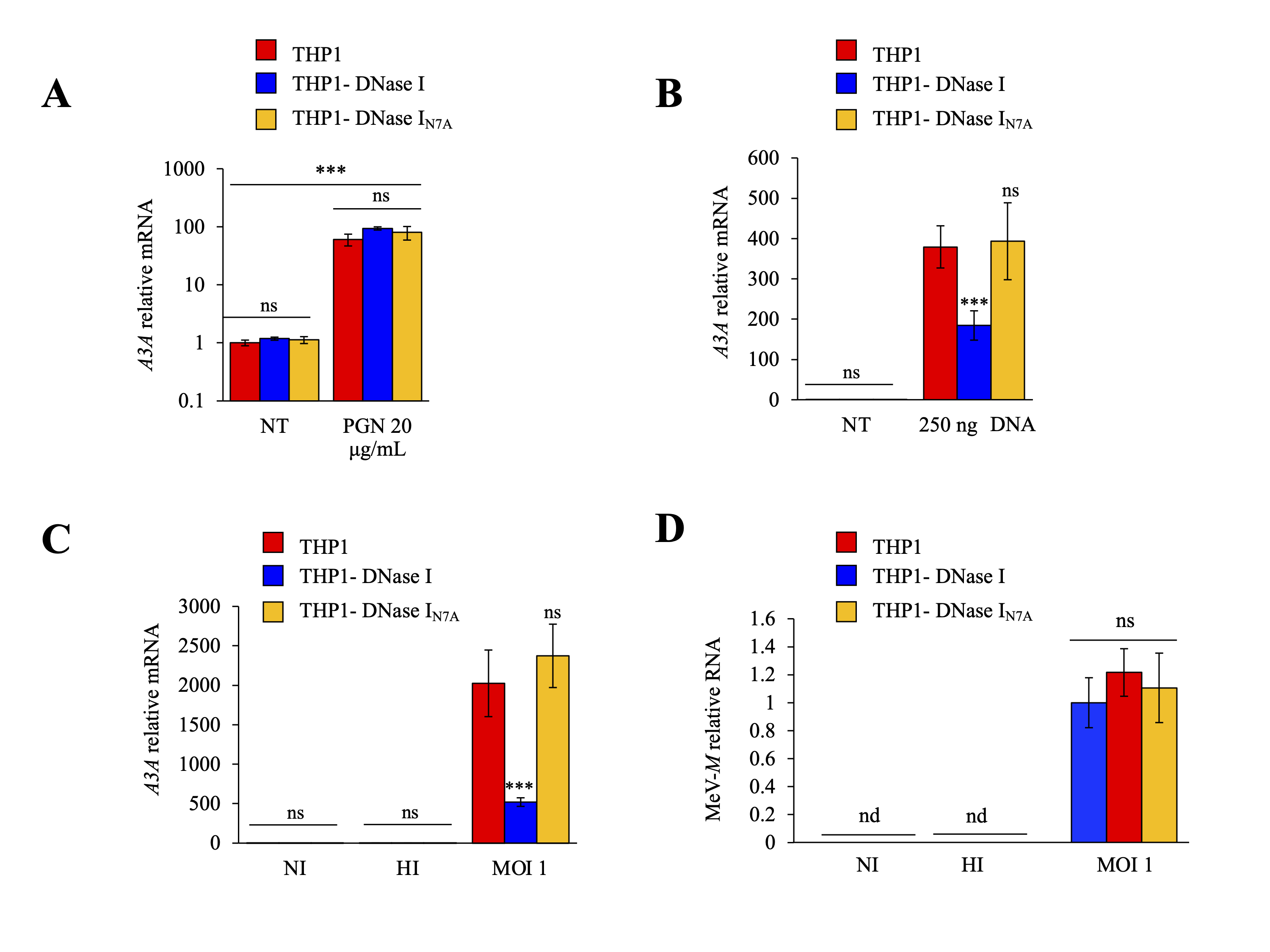

Supplement: S5 Fig — A) APOBEC3A profiling by RT-qPCR at 24 hours by incubating THP-1 with 20 μg/ml of PGN. B) APOBEC3A profiling by RT-qPCR at 24h post-tranfection of 250 ng of DNA. C) APOBEC3A profiling by RT-qPCR at 24 hpi with MeV at MOI 1 infection in THP-1 expressing DNase I or not, negative control was performed with a heat inactivated (HI) virus. Control was performed. Data were normalized to the RPL13A housekeeping gene. D) MeV M RNA profiling by RT-qPCR at 24 hpi in stable DNase I or a catalytically inactive mutant DNase IN7A overexpressed THP-1 cell lines. A-D) Mean values and s.e.m. were calculated for three independent experiments in duplicate (n = 6), unpaired two-side Student’s t-test, ***, p < 0.005 and ns: not statistically significant. (TIFF) [file ppat.1011170.s005.tiff]

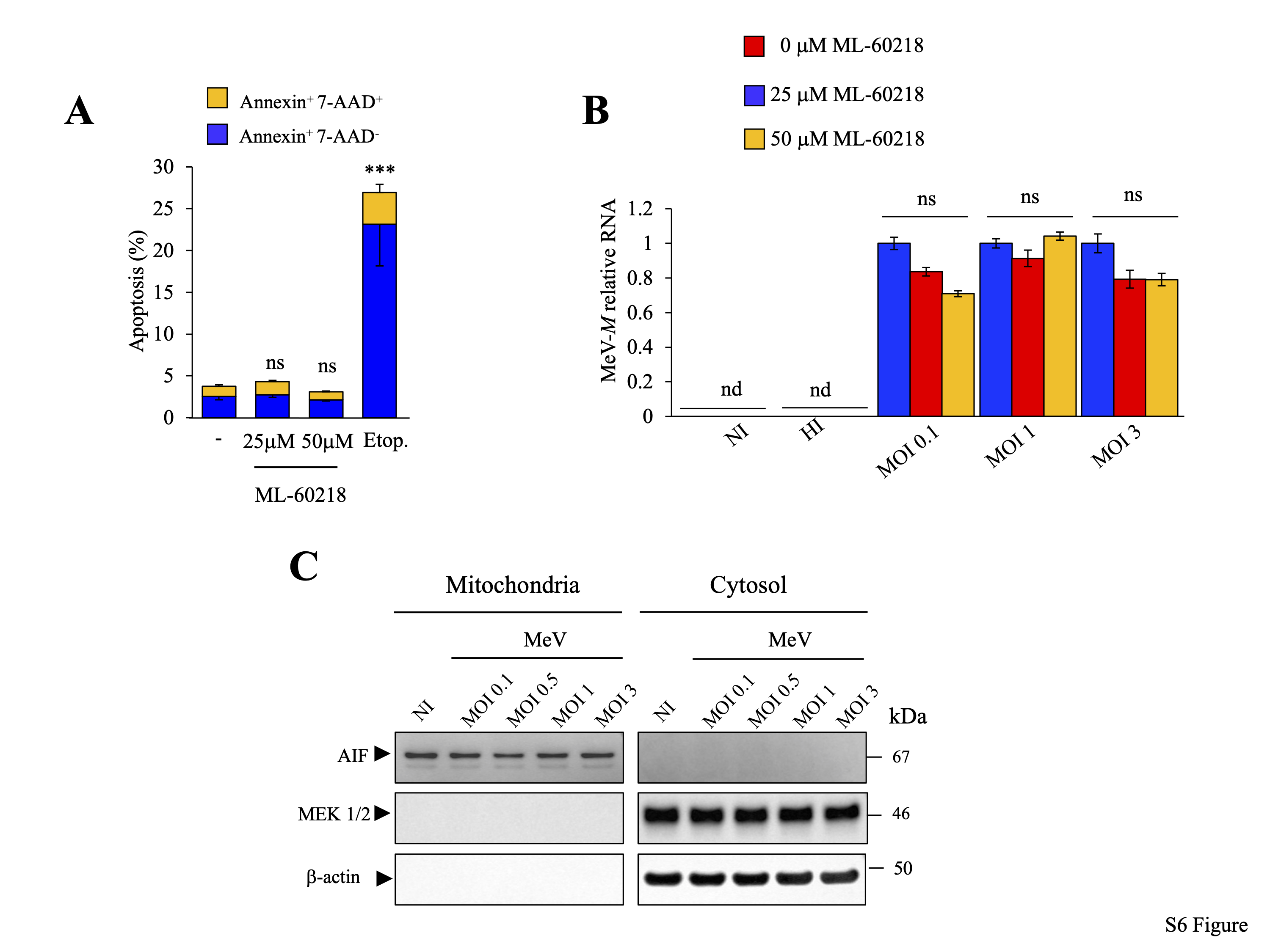

Supplement: S6 Fig — A) Flow cytometry analysis of early apoptosis (annexin V+ 7-AAD− cells) and late apoptosis/necrosis (annexin V+ 7-AAD+ cells) in HeLa cells incubated with 25μM and 50μM of ML-60218. Positive control was performed by incubating with Etoposide. The error bars represent the s.d. from three independent experiments using a two-way analysis of variance (ANOVA test). B) MeV M mRNA profiling by RT-qPCR at 24 hpi in THP-1 treated with 25μM or 50μM of ML-60218, negative control with a heat inactivated (HI) virus. Data were normalized to the RPL13A housekeeping gene. Mean values and s.e.m. were calculated for three independent experiments in duplicate (n = 6), unpaired two-side Student’s t-test, ***, p < 0.005 and ns: not statistically significant, nd: not determined. C) Purity control of cellular fractions by immunoblotting, using β-actin to indicate the cytosol, AIF (apoptosis inducing factor) for mitochondria and MEK1/2 as a marker of cytosolic proteins. kDa: kilo Dalton. (TIFF) [file ppat.1011170.s006.tiff]

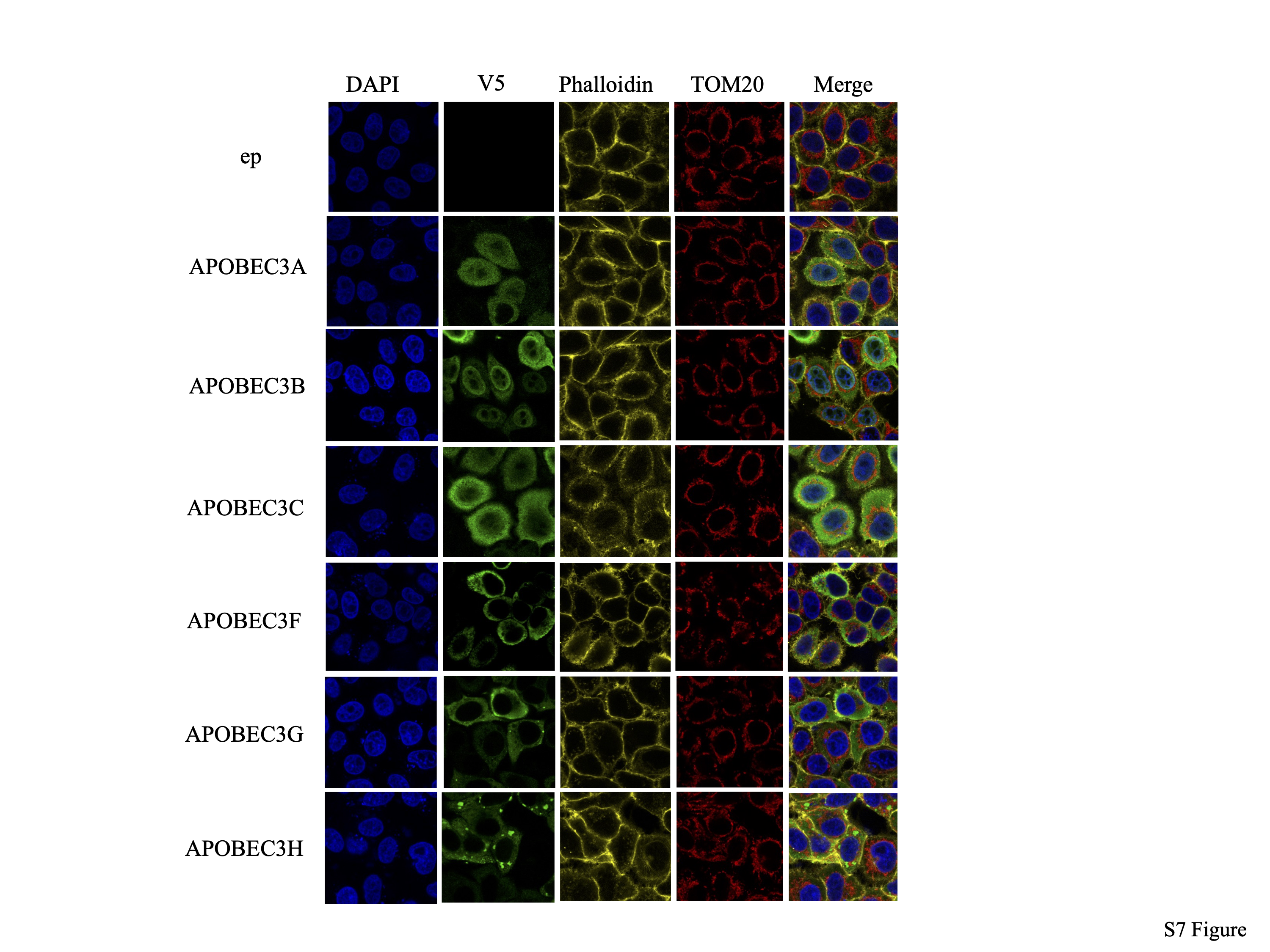

Supplement: S7 Fig — Confocal microscopy of V5-tagged A3A-A3H plasmids performed in HeLa cells at 24 hours post transfection. Nuclei are stained using DAPI (blue), and mitochondrial network detection was performed with anti-TOM20 (red), APOBEC3 with anti-V5 (green), and actin was visualized using phalloidin (yellow). ep: empty plasmid. (TIFF) [file ppat.1011170.s007.tiff]
