# Supplementary material for: Antagonism of ALAS1 by the Measles Virus V protein contributes to degradation of the mitochondrial network and promotes interferon response
Source: PLoS Pathog. 2023 Feb 21;19(2):e1011170. doi: 10.1371/journal.ppat.1011170 (PMC9983871; doi:10.1371/journal.ppat.1011170)
Supplement: S1 Table — fwd: forward, rev: reverse. (DOCX) [file ppat.1011170.s008.docx]

**Primer Sequences Probes**

________________________________________________________________________________________________

*Rig-I (DDX58)* fwd 5’CTTTTTCTCAAGTTCCTGTTGGA *UPL79*

*Rig-I (DDX58)* rev 5’TCCCAACTTTCAATGGCTTC

*HPRT1* fwd 5’TGACCTTGATTTATTTTGCATACC *UPL73*

*HPRT1* rev 5’CGAGCAAGACGTTCAGTCCT

*IFN-α* fwd 5’TCCTGCTTGAAGGACAGACA *UPL63*

*IFN-α* rev 5’TTTCAGCCTTTTGGAACTGG

*IFN-β* fwd 5’CTTTGCTATTTTCAGACAAGATTCA *UPL20*

*IFN-β* rev 5’GCCAGGAGGTTCTCAACAAT

*MDA5* fwd 5’GGCACCATGGGAAGTGATT *UPL20*

*MDA5* rev 5’GATGATGATATTCTTCCCTTCCA

*RNA pol III* fwd 5’GCTGGACAAGAGCAACAGC *UPL21*

*RNA pol III* rev 5’TCATCTGTGATATGTTAATGAAGGAAC

*qRPL13* fwd 5’ctggaccgtctcaaggtgtt *UPL74*

*qRPL13* rev 5’gccccagataggcaaactt

*β2M* fwd 5’AATCAGATGGGTGTAGATCAAGG *UPL15*

*β2M* rev 5’GTTTCCACCCCTTCCATTTT

*dHCoxI* fwd 5’GCGGTTGACTATTCTCTACAAACCACAAA

*dHCoxI* rev 5’GGGGGTTTTATATTGATAATTGTTGTGATGAAA

*MeV-M* fwd 5’CACCACCTACAGTGATGGCAGGCTGGT

*MeV-M* rev 5’AACACGGAACCTCTGCGGGGTATCGAG

*MeV-H-L* fwd 5’TCAGGCATACCCACTAGTGTGAA

*MeV-H-L* rev 5′TGACAGATAGCGAGTCCATAACG

*MeV-H-L* probe 5′FAM-CATCAGAATTAAGAAAAACGTAG-TAMRA

*β-actin* fwd 5’ACCGAGCGCGGCTACAG

*β-actin* rev 5′CTTAATGTCACGCACGATTTCC

*β-actin* probe 5′FAM-CACCACCACGGCCGA-TAMRA

_______________________________________________________________________________________________

**S1 Table**

Compendium of primers used for PCR, qPCR and RT-PCR; qPCR was either done by SYBR green or TaqMan with given UPL probes.

Fwd: forward, rev: reverse.
